# Epigenetic reprogramming of cell cycle genes by ACK1 promotes breast cancer resistance to CDK4/6 inhibitor

**DOI:** 10.1038/s41388-023-02747-x

**Published:** 2023-06-17

**Authors:** Mithila Sawant, Audrey Wilson, Dhivya Sridaran, Kiran Mahajan, Christopher J. O’Conor, Ian S. Hagemann, Jingqin Luo, Cody Weimholt, Tiandao Li, Juan Carlos Roa, Akhilesh Pandey, Xinyan Wu, Nupam P. Mahajan

**Affiliations:** 1 Department of Surgery, Washington University in St. Louis, Cancer Research Building, 660 Euclid Ave., St. Louis, MO 63110 USA; 2 Division of Urologic Surgery, Washington University in St. Louis, Cancer Research Building, 660 Euclid Ave., St. Louis, MO 63110 USA; 3 Department of Pathology and Immunology, Washington University in St. Louis, Cancer Research Building, 660 Euclid Ave., St. Louis, MO 63110 USA; 4 Siteman Cancer Center, Washington University in St. Louis, Cancer Research Building, 660 Euclid Ave., St. Louis, MO 63110 USA; 5 Bioinformatics Research Core, Center of Regenerative Medicine, Department of Developmental Biology, Washington University in St. Louis, St. Louis, MO 63110 USA; 6 Department of Pathology, Pontificia Universidad Católica de Chile, Santiago, Chile; 7 Department of Laboratory Medicine and Pathology, Mayo Clinic, Rochester, MN 55905 USA; 8 Molecular Pharmacology and Experimental Therapeutics, Mayo Clinic, Rochester, MN 55905 USA

**Keywords:** Cancer genomics, Gene silencing, Chemotherapy

## Abstract

Hormone receptor-positive, HER2-negative advanced breast cancers exhibit high sensitivity to CDK4/6 inhibitors such as palbociclib. However, most patients inevitably develop resistance, thus identification of new actionable therapeutic targets to overcome the recurrent disease is an urgent need. Immunohistochemical studies of tissue microarray revealed increased activation of non-receptor tyrosine kinase, ACK1 (also known as TNK2) in most of the breast cancer subtypes, independent of their hormone receptor status. Chromatin immunoprecipitation studies demonstrated that the nuclear target of activated ACK1, pY88-H4 epigenetic marks, were deposited at cell cycle genes, *CCNB1, CCNB2* and *CDC20*, which in turn initiated their efficient transcription. Pharmacological inhibition of ACK1 using its inhibitor, (*R*)-**9b** dampened *CCNB1, CCNB2* and *CDC20* expression, caused G2/M arrest, culminating in regression of palbociclib-resistant breast tumor growth. Further, (*R*)-**9b** suppressed expression of CXCR4 receptor, which resulted in significant impairment of metastasis of breast cancer cells to lung. Overall, our pre-clinical data identifies activated ACK1 as an oncogene that epigenetically controls the cell cycle genes governing the G2/M transition in breast cancer cells. ACK1 inhibitor, (*R*)-**9b** could be a novel therapeutic option for the breast cancer patients that have developed resistance to CDK4/6 inhibitors.

## Introduction

Breast cancer (BC) is the most common cancer in women with significant cancer related deaths worldwide [1]. Estrogen receptor (ER), a nuclear hormone receptor regulates the expression of distinct set of genes; a large majority of BCs are estrogen receptor (ER)-positive, of which about 65% are also progesterone receptor (PR)-positive [2]. While most ER^+^ breast cancer may initially respond to hormone therapy (also called endocrine therapy), 15–20% of tumors are intrinsically resistant to treatment, and another 30–40% acquire resistance to treatment over a period of many years. Multiple mechanisms have been shown to be responsible for resistance including, deregulated expression of ER and its co-activators/repressors [3], aberrant receptor/non-receptor tyrosine kinase activity, high mutation rate and epigenetic alterations of *ESR1* gene that encodes for ER [4–6] and expression of truncation variants [7], along with deregulation of cell cycle [8, 9]. On the other hand, due to the lack of any particular molecular drivers, targeting TNBCs has emerged to be a major challenge. Currently, cytotoxic chemotherapy is employed to treat TNBC patients [10], however, the high level of chromosomal instability exhibited by TNBCs attribute an aggressive and more resilient phenotype culminating in an early relapse [11, 12]. Overall, inevitable resistance to current therapies warrants the development of new targets and therapeutic agents that overcome tumor growth-driving signaling pathways.

CDK4 and CDK6 are cell cycle kinases that work in complex with cyclin D1 to phosphorylate tumor suppressor retinoblastoma protein (Rb). Rb-phosphorylation inhibits its activity resulting in dissociation from E2F transcription factors, which activate genes involved in DNA replication and cell cycle progression. Frequent deregulation of the cyclin D‐CDK4/6‐INK4‐RB pathway in breast cancer has led to development of CDK4/6 inhibitors, palbociclib (PD0332991), ribociclib (LEE011), trilaciclib and abemaciclib (LY2835219) [13, 14]. CDK4/6 inhibitors control tumor growth by blocking G1 to S cell cycle transition in cancer cells and have been used for treatment of recurrent ER-positive, HER2-negative metastatic breast cancer [15, 16]. Lately, these FDA approved drugs have been pre-clinically and clinically tested in multiple cancers [17]. Although they exhibited promising clinical outcomes, intrinsic or acquired resistance to CDK4/6 inhibitors by amplification of the CDK6 kinase [18], and employing other cyclins in G1 phase, has culminated in early adaptation and resistance [18, 19]. Combining CDK inhibitors with endocrine therapy although improved the outcomes, the resistant tumors showed accelerated loss of estrogen signaling with convergent upregulation of JNK signaling through growth factor receptors, while those maintaining estrogen signaling showed ERK upregulation through ERBB4 signaling [20]. Together these data indicate that a shift from estrogen to alternative growth factor signaling plays a critical role in resistance, which necessitates the engagement of therapies targeting protein/s governing these signaling pathways.

ACK1 (TNK2) is an oncogenic non-receptor tyrosine kinase, over-expressed in multiple cancers such as prostate [21–25], breast [26–28], NSCLC [29, 30], glioma [31], ovarian [30], pancreatic [32] and colorectal cancer [33]. The tumor cell’s ACK1 addiction is supplemented by its gene-amplification, mutation and post-translational modifications, resulting in its hyper-phosphorylation, primarily at Tyr284, and kinase activation thereby aiding tumor progression and metastasis [34]. ACK1 is a downstream effector of multiple receptor tyrosine kinases e.g., EGFR, MERTK, HER2, PDGFR and insulin receptor [27, 35, 36] and relays extracellular signals to downstream effectors such as AKT, phosphorylation of which at Tyr176, lead to its activation independent of the PI3K pathway [27, 37]. Emerging evidences indicate epigenetic function of ACK1 could be involved in driving drug-resistance; ACK1 directly phosphorylated histone H4 at Tyr88 (pY88-H4), orchestrating global androgen receptor (AR) transcription program [21]. ACK1 mediated AR Tyr-phosphorylation was also shown to be critical for androgen receptor or AR Lys609-acetylation contributing toward Enzalutamide resistance [22]. Recently, another target of ACK1 was identified; it phosphorylated C-terminal Src kinase (CSK) at Tyrosine 18, which enhanced CSK function, constraining T-cell activation [38]. In addition, inhibition of ACK1 with its inhibitor, (*R*)-**9b** synergized with osimertinib in inhibiting the growth of EGFR mutant NSCLC cell lines [29]. Moreover, histone deacetylase inhibitor, entinostat (MS-275) attenuated ACK1 in kidney cancer cells [39]. Although, these evidences point toward the important contribution of ACK1 in various malignancies, neither the targets of ACK1 epigenetic activity, nor their functional relevance in breast cancer is fully understood. We uncovered that ACK1 is activated in four different subtypes of the breast cancer and epigenetically alters the chromatin landscape associated with genes involved in G2/M transition of cell cycle. Further, we demonstrate that its functional obstruction alleviates tumor progression, including re-sensitization of palbociclib-resistant breast tumor cells, opening a new therapeutic modality for the patient with the recurrent disease.

## Results

### ACK1 activation in multiple subtypes of breast cancers

**Fig. 1:**
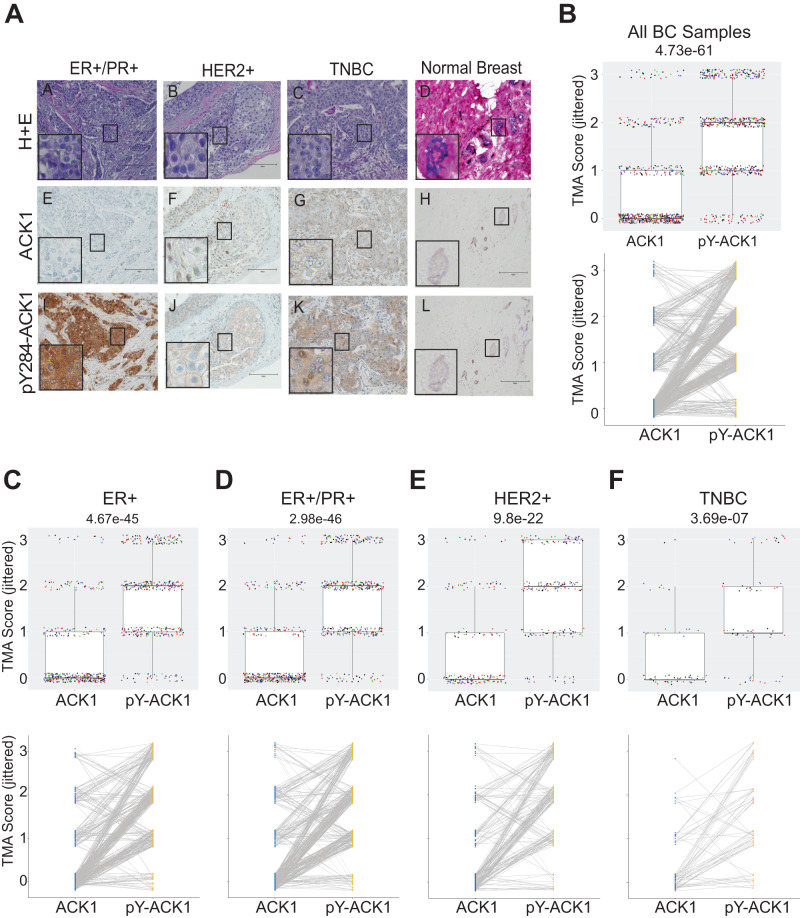
ACK1 is activated in multiple types of breast cancers. **A** Immunohistochemical analysis of human breast cancer tissue samples and normal parenchyma was performed using pY284-ACK1 and total ACK1 antibodies. Tumor status was confirmed by H&E staining (*n* = 500 all groups; a representative image is shown). Relative pairwise abundance of total ACK1 and pY284-ACK1 displayed by TMA staining for (**B**) all breast cancer samples, (**C**) ER^+^, (**D**) ER^+^/PR^+^, (**E**) HER2^+^, and (**F**) TNBC samples is shown.

To obtain a comprehensive understanding of ACK1 activity, we generated four distinct breast tissue microarrays (TMAs) comprising of different grades and molecular subtypes of breast cancer samples from over 400 breast cancer patients. Immunohistochemical (IHC) analysis revealed a moderate to strong ACK1 activation (pY284-ACK1) in 57% (53/92) of ER^+^, 58% (112/192) of ER^+^/PR^+^, 70% (50/72) of HER2^+^, and 47% (44/94) of TNBC breast cancer samples (Fig. 1A and Supplementary Table S1). A significant increase in pY284-ACK1 expression was seen in all the four types of breast cancers that were examined (Fig. 1B, C–F, top panels). When pairwise comparison was performed, most breast cancer patients exhibited significantly higher pY284-ACK1 levels as compared to total ACK1 in all the four types of breast cancers (Fig. 1C–F, lower panels), suggesting that it is activated ACK1, not the total ACK1, that is selectively elevated in majority of the breast tumors. cBioPortal cancer genomics reveals that out of 379 metastatic breast cancer cases, 116 exhibited *ACK1*/*TNK2* gene amplification (30.6%), while just one sample showed mutation (S588Y) in *ACK1* gene, indicating that the gene amplification could be the major cause of the ACK1 activation in breast cancers (Supplementary Fig. S1). The ACK1 gene amplification was seen in ER^+^ (21/116), ER^+^/PR^+^ (13/116), HER2^+^ (15/116), and TNBCs (10/116) (Supplementary Fig. S1). Overall, these data opened up an intriguing possibility that a significant proportion of four distinct subtypes of breast cancers exhibit ACK1 activation, which is independent of their hormone receptor status.

### ACK1 inhibitor (*R*)-9b has drug-like properties

**Fig. 2:**
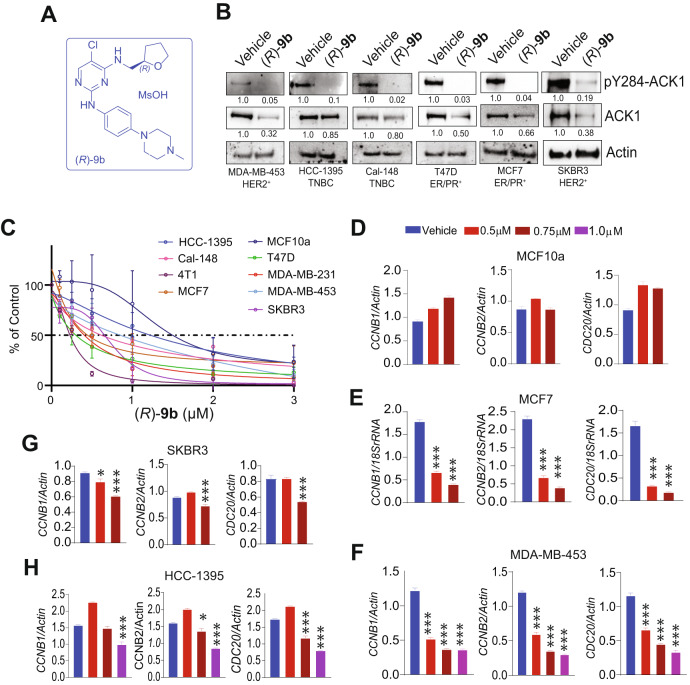
ACK1 regulates breast cancer transcriptome, affecting genes implicated in cell cycle progression. **A** Chemical structure of (*R*)-**9b**. **B** Breast cancer cell lines were treated with either vehicle or (*R*)-**9b** overnight and cell lysates were subjected to immunoprecipitation using ACK1 antibody, followed by immunoblotting with pY284-ACK1 antibody. Lysates were also subjected to immunoblotting with total ACK1 and Actin antibodies, shown in the lower panels. Densitometric measurement of protein abundance relative to control is displayed below each blot. **C** Breast cancer cell lines were treated with varying concentrations of (*R*)-**9b** for 96 h and the cell proliferation was measured using Trypan Blue exclusion method. **D**–**H** RNA from breast cancer cells treated with vehicle or varying concentrations of (*R*)-**9b** overnight, and were subjected to qRT-PCR using *CCNB1*, *CCNB2* and *CDC20* primers. *Actin* or *18S rRNA* was used as housekeeping control. For (**B**), representative images of three independent experiments are shown. For (**C**), a four parameter, variable slope, non-linear regression curve was computed, representing three independent experiments. For (**D**–**H**), data are represented as the mean ± SEM. *p < 0.05, **p < 0.01, ***p < 0.001, unpaired two-tailed Student’s *t* test.

Presence of activated ACK1 across multiple molecular subtypes suggests that ACK1-targeted therapies may be effective not only in ER^+^, ER^+^/PR^+^ and HER2^+^ tumors, but can also be potentially exploited across other subtypes, especially the TNBCs where lack of clearly defined therapeutic targets has resulted in significant mortality. We developed a new class of ACK1 small molecule inhibitor, (*R*)-**9b** (Fig. 2A) and many of its derivatives. These were subjected to in vitro kinase assay revealing that (*R*)-**9b** is the most potent ACK1 inhibitor with IC_50_ of 13 nM (Supplementary Table S2). The mesylate salt, referred as (*R*)-**9b**MS was highly soluble in aqueous media and exhibited high membrane permeability in Caco-2 assay (Supplementary Table S3). In vitro studies with human liver microsomes showed that (*R*)-**9b** had a low potential to inhibit cytochrome P450 (CYP) isoenzymes, including those that are most relevant for drug metabolism in humans (CYP1A1/2, CYP2C9, CYP2C19, CYP2D6 and CYP3A4) (Supplementary Table S4). The distribution of drug from plasma to target tissues can be effected by its Plasma Protein Binding (PPB). Compounds that are extensively bound to plasma proteins will have a low volume of distribution (VDss), can have long plasma half-lives (T_1/2_), and have low clearance (Cl) by both liver (hepatic) and kidney (renal) routes, and thus impacting the efficacy. (*R*)-**9b** exhibited high PPB (83%) and over 96% was remaining after 6 h of treatment (Supplementary Table S5). Further, (*R*)-**9b** exhibited good stability in gastric and intestinal fluids, indicating that it can be orally administered (Supplementary Table S6).

Assessing the specificity of lead compounds early in development using highly relevant and predictive assays allows more informed decisions about compound safety, ultimately leading to the development of safer and more effective drugs. We performed pharmacological profiling of (*R*)-**9b**, to identify undesirable off-target activity profile using DiscoverX SAFETYscan, which has a broad menu containing 78 assays, and provides functional data for cell surface receptors, ion channels, kinases, nuclear hormone receptors, GPCRs and other relevant enzymes, inhibition of which could cause significant toxicity in human clinical trials. None of the 78 targets were inhibited significantly by (*R*)-**9b** (Supplementary Table S7). Consistent with these data, (*R*)-**9b** exhibited high potency against various prostate cancer cell lines (IC_50_ 0.4 µm) and xenograft tumors, with no undesirable side effects [21]. Taken together, these data indicates that (*R*)-**9b** is a ‘safe compound’ with good drug-like properties.

### ACK1 inhibition by (*R*)-9b induced apoptosis in breast cancer cells

To examine the expression of activated ACK1 and its sensitivity to (*R*)-**9b**, various breast cancer cell lines, including TNBC cell lines (MDA-MB-231, HCC-1395 and Cal148), ER^+^/PR^+^ cell lines (T47D and MCF7) and HER2^+^ cell lines (SKBR3 and MDA-MB-453,) were subjected (*R*)-**9b** treatment, followed by immunoblotting with pY284-ACK1 antibodies. All of these cell lines exhibit significant ACK1 activation, which was compromised upon (*R*)-**9b** treatment as evidenced by the reduced pY284-ACK1 levels irrespective of their hormone receptor status (Fig. 2B). The breast cancer cell lines treated with (*R*)-**9b** exhibited a significant decrease in proliferation in a concentration dependent manner, with the IC_**50**_ between 0.25–1.22 µM, in contrast, normal breast cell line, MCF10A exhibited IC_50_ of 1.49 µM (Fig. 2C and Supplementary Table S8). Inhibition of ACK1 using (*R*)-**9b** in MCF7, SKBR3, HCC-1395 and MDA-MB-453 induced a marked increase in the number of cells undergoing apoptosis (Supplementary Fig. 2A, B), indicating the susceptibility of the breast cancer cells to ablation of ACK1 kinase activity.

### ACK1 regulates breast cancer transcriptome, affecting genes implicated in cell cycle progression

Since (*R*)-**9b** treatment compromised proliferation of four distinct subtypes of breast cancers, it opened up the possibility that activated ACK1 signaling-dependent but hormone-independent mechanism might be executed by breast cancer cells for its proliferation. To unveil these potential downstream players of activated ACK1, we explored the outcome of its inhibition on breast cancer transcriptome by performing RNA-sequencing in two cell lines, MDA-MB-453 and HCC-1395. KEGG analysis revealed that the topmost modulated genes belonged to the cell cycle pathway (Supplementary Fig. S3A, B and Supplementary Table S9). Integrating the RNA-seq data (Supplementary Fig. S3C) and pY88-H4 ChIP-Seq data (described below) (Supplementary Fig. S4A), we focused on cell cycle regulating genes *CDC20, CCNB1* and *CCNB2*. In order to estimate the effect of ACK1 inhibition, breast cell lines were treated with (*R*)-**9b**, followed by qRT-PCR. As anticipated, a significant downregulation of these mRNA transcripts was seen in MCF7, MDA-MB-453, SKBR3, and HCC-1395 cells, but not in normal MCF10a cells (Fig. 2D–H).

**Fig. 3:**
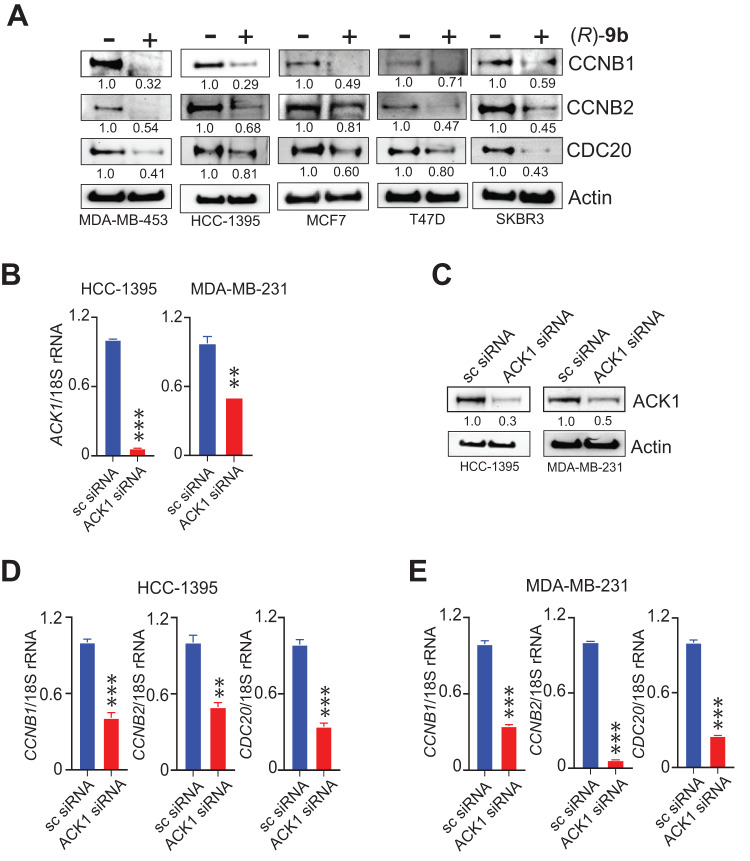
ACK1 inhibitor suppresses expression of cell cycle genes. **A** Cell lysates from vehicle or (*R*)-**9b** treated cells were immunoblotted using CCNB1, CCNB2 and CDC20 antibodies. **B** HCC-1395 and MDA-MB-231 cells were transfected with ACK1 and scrambled siRNAs, followed by qPCR using ACK1 primers, with 18S rRNA as internal control. **C** siRNA transfected cells were subjected to western blotting using ACK1 antibody, with Actin as loading control. RNA from silenced cells, (**D**) HCC-1395 and (**E**) MDA-MB-231 was subjected to qPCR using *CCNB1*, *CCNB2* and *CDC20* primers. 18S rRNA was used as housekeeping control. For (**A**) and (**C**), representative images of three independent experiments are shown. For (**B**, **D**, **E**), data are represented as the mean ± SEM. *p < 0.05, **p < 0.01, ***p < 0.001, unpaired two-tailed Student’s *t* test.

The loss of transcription was also reflected in decrease in respective protein abundance upon (*R*)-**9b** treatment (Fig. 3A). The role of ACK1 on the cell cycle regulating genes was further validated by its silencing in HCC-1395 and MDA-MB-231 cell lines. ACK1 knockdown significantly inhibited the transcription of *CCNB1*, *CCNB2* and *CDC20* (Fig. 3B–E).

### Activated ACK1 epigenetically regulates cell cycle genes by depositing pY88-H4 marks

**Fig. 4:**
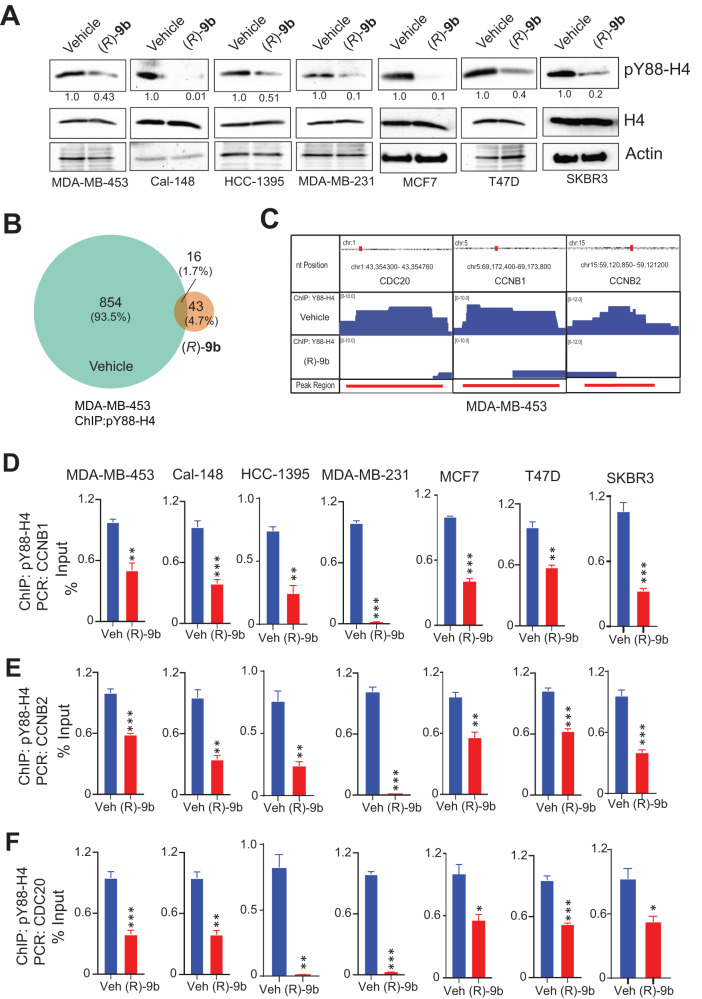
ACK1 epigenetically modifies the chromatin landscape by depositing pY88-H4 activating marks, regulating cell cycle genes. **A** Breast cancer cell lines were treated with (*R*)-**9b** overnight and the cell lysates were subjected to immunoprecipitation using pY88-H4 antibody, followed by immunoblotting with H4 antibody. Lysates were also subjected to immunoblotting with total H4 and Actin antibodies, shown in the lower panels. Densitometric measurement of protein abundance relative to control is displayed below each blot. **B** Venn diagrams summarizing the overlap between sites bound by pY88-H4 in vehicle treated and (*R*)-**9b** treated MDA-MB-453 cells. **C** ChIP-Seq using pY88-H4 antibody in MDA-MB-453 revealed peaks in *CCNB1*, *CCNB2* and *CDC20* genes. Breast cancer cells were subjected to ChIP using pY88-H4 antibody followed by qPCR for (**D**) *CCNB1*, (**E**) *CCNB2*, and (**F**) *CDC20* genes. For (**A**), representative images of three independent experiments are shown. For (**D**–**F**), data are represented as the mean ± SEM. *p < 0.05, **p < 0.01, ***p < 0.001, unpaired two-tailed Student’s *t* test.

Epigenetic reprogramming of the chromatin landscape by epigenetic drivers has come to the forefront as a crucial conductor for drug-resistance, implicating the urgency for unraveling new epigenetic targets. ACK1 kinase acts as an epigenetic modulator by phosphorylating histone H4 at Y88, leading to WDR5/MLL2 complex recruitment and activation of androgen receptor (AR) transcription [21]. This ChIP-seq data also revealed deposition of pY88-H4 marks in *CDC20*, *CCNB1* and *CCNB2* genes (Supplementary Fig. S4A). We reasoned that ACK1’s epigenetic activity may be the prime reason behind the significant increase in transcriptional output of the cell cycle genes. To explore this possibility, we examined our previous mass spectrometry analysis on the phosphotyrosine proteomes of breast cancer cell lines [26, 40] and found pY88-H4 epigenetic event in 5 BC cell lines, BT549, HCC1178, MDA-MB-435, MDA-MB-436 and SUM149 (Supplementary Fig. S4B, C). To further validate deposition of pY88-H4 marks by activated ACK1, breast cancer cell lines were treated with (*R*)-**9b**, followed by immune-blotting. A significant expression of Y88-H4 epigenetic marks was observed in all the cell lines, which was significantly compromised upon (*R*)-**9b** treatment (Fig. 4A).

To examine the transcription program regulated by pY88-H4, chromatin prepared from vehicle- or (*R*)-**9b**-treated MDA-MB-453 cells was immunoprecipitated (ChIP) with pY88-H4 antibody, followed by next-generation sequencing (ChIP-seq). Peak analysis revealed pY88-H4 binding to 935 sites (in 854 genes) (Supplementary Table S10). The pY88-H4 peaks were primarily annotated in intergenic regions and introns (Supplementary Table S11A), mainly in protein coding genes (Supplementary Table S11B). Further, Venn diagram (VD) analysis revealed an occupancy of pY88-H4 at 854 unique sites, with 16 sites shared between vehicle- and (*R*)-**9b**-treated cells (Fig. 4B). The ChIP-seq of MDA-MB-453 cells also revealed deposition of pY88-H4 marks in *CDC20, CCNB1* and *CCNB2* genes (Fig. 4C). The pY88-H4 binding motifs predicted by HOMER, including the corresponding relative score, sequence, and transcription factors are shown in Supplementary Fig. S5A, which show a distinct set of 3 Motifs used by pY88-H4. The predicted gene ontology (GO) molecular pathways and the KEGG pathway analysis hits are shown in Supplementary Fig. S5B, C, respectively.

In order to validate deposition of these marks, breast cancer cell lines were subjected to ChIP using pY88-H4 antibody, followed by qPCR for the loci corresponding to peaks for *CDC20, CCNB1* and *CCNB2*. It revealed the presence of pY88-H4 marks at these gene loci, which were significantly compromised upon (*R*)-**9b** application (Fig. 4D–F).

### (*R*)-9b induces G2/M phase cell cycle arrest and overcomes CDK4/6 inhibitor resistance

**Fig. 5:**
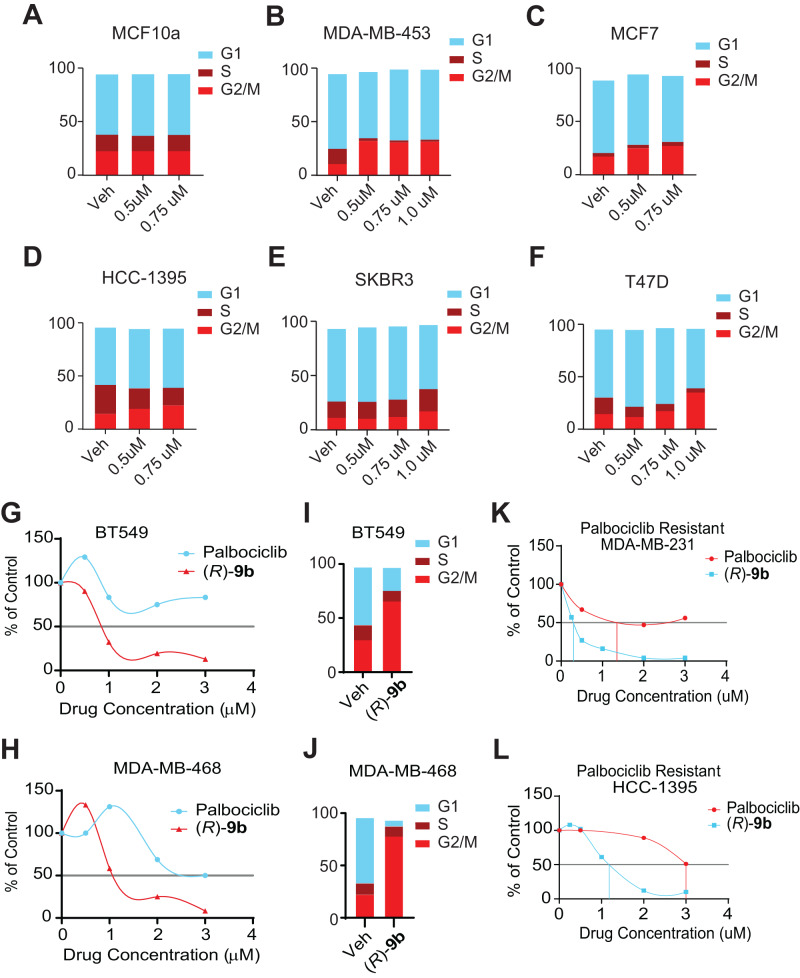
ACK1 inhibition leads to G2/M cell cycle arrest in breast cancer cells. **A**–**F** Breast cancer cells were treated with either vehicle or (*R*)-**9b** for 48 h; cells were harvested and processed for cell cycle analysis using propidium iodide staining, followed by flow cytometry. **G** BT549 and **H** MDA-MB-468 cells were treated with varying drug concentrations of palbociclib or (*R*)-**9b** for 96 h and the cell viability assessed using trypan blue exclusion method. **I** BT549, and **J** MDA-MB-468 cells were treated with 1 µM palbociclib or (*R*)-**9b** for 48 h and subjected to cell cycle analysis using propidium iodide staining, followed by flow-cytometry. Palbociclib-resistant MDA-MB-231 (**K**) and HCC-1395 (**L**) were generated and treated with varying drug concentrations of palbociclib or (***R***)-**9b** for 96 h and the cell viability assessed using trypan blue exclusion method.

*CDC20* is required for anaphase and chromosome separation, whereas *CCNB1* and *CCNB2* are essential for early events of mitosis [41–44], suggesting that compromising expression of these genes could lead to cell cycle arrest. Cell cycle analysis was employed to assess the cumulative effect of ACK1 inhibition and suppression of cell cycle gene transcription, which revealed arrest in G2/M phase upon (*R*)-**9b** treatment in breast cancer cell lines, MDA-MB-453, MCF7, HCC-1395, SKBR3 and T47D, however, no G2/M arrest was noticed in normal breast cells, MCF10a (Fig. 5A–F). Further, silencing of ACK1 in HCC-1395 and MDA-MB-231 cells too led to a G2/M arrest in these cells, validating the role of ACK1 in the progression of G2/M phase of the cell cycle (Supplementary Fig. S6A, B).

The mid-G1 phase is governed by CDK4 and CDK6, two serine/threonine kinases the catalytic activity of which is modulated by D-type cyclins (D1, D2, and D3) [45]. The cyclin D-CDK4/6 complex selectively phosphorylates and inactivates members of Retinoblastoma-associated proteins (pRb), such as p110 (encoded by *RB1*), the related pocket protein p107 (encoded by *RBL1*) and p130 (encoded by *RB2*) [46]. The CDK inhibitors inhibit the downstream CDK4/6-mediated phosphorylation of Rb, which although potently arrest cell cycle, requires functional Rb protein [47]. Since the cell cycle analysis revealed that the ACK1 inhibition cause G2/M arrest, we reasoned that CDK4/6 inhibitors (palbociclib, ribociclib and abemaciclib) resistant cells that overcome G1 arrest could now be sensitized by using (*R*)-**9b** treatment, causing G2/M arrest. To address this possibility, cell proliferation of cell lines intrinsically lacking Rb (MDA-MB-468, and BT549) and thus are resistant to palbociclib, was examined. Both BT549 and MDA-MB-468 cell lines were observed to be sensitive to (*R*)-**9b** treatment (Fig. 5G, H), indicating breast cancer cells resistant to CDK4/6 inhibitors can be rendered susceptible to ACK1 inhibition. (*R*)-**9b** was also successful in inducing a G2/M cell cycle arrest in the palbociclib-resistant BT549 and MDA-MB-468 cells (Fig. 5I, J). To further validate, MDA-MB-231 and HCC-1395 cells, which are palbociclib-sensitive, were grown in presence of palbociclib and selected to establish the resistant lines. These palbociclib-resistant cells lines were sensitive to (*R*)-**9b** and exhibited loss of proliferation (Fig. 5K, L).

### ACK1 inhibitor (*R*)-9b curbs breast tumor growth

**Fig. 6:**
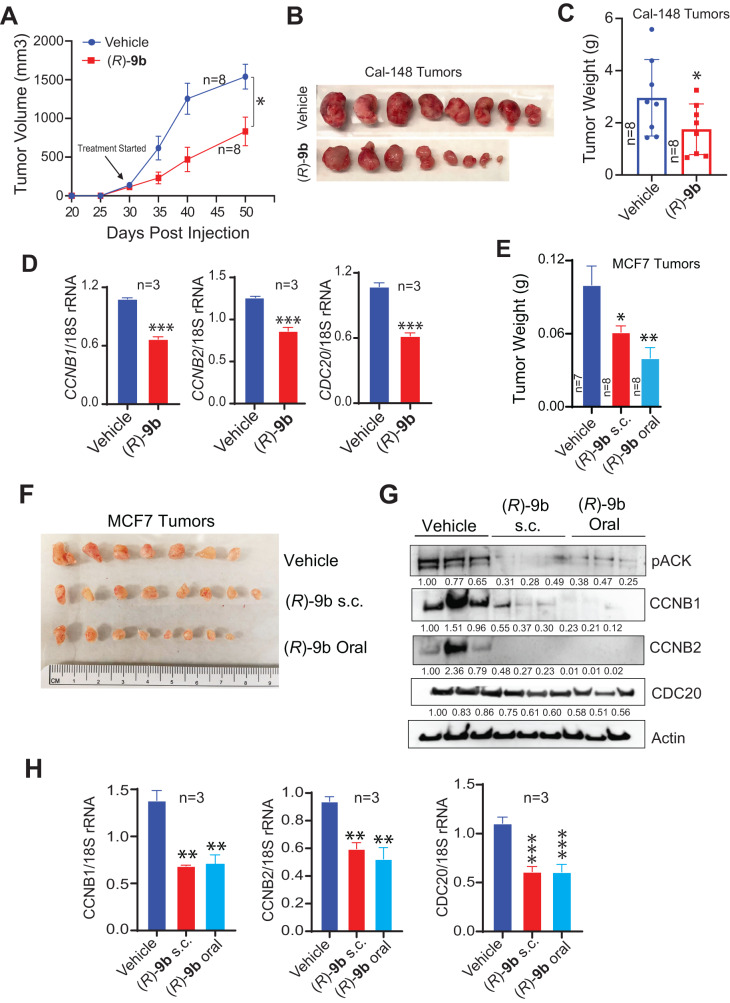
(*R*)-9b inhibits breast cancer xenograft tumor growth in vivo. **A** 2 × 10^6^ Cal-148 cells were injected in the 4th inguinal mammary fat pad of female SCID mice. Once the tumors were palpable, the mice were treated subcutaneously with either vehicle (Captisol; *n* = 8) or (*R*)-**9b** at 24 mg/Kg (*n* = 8) five times a week, for 4 weeks. The tumor volumes were measured twice a week. **B**, **C** The tumors were harvested and photographed, and the weights were recorded. **D** RNA was prepared from the tumors, followed by qRT-PCR to determine the levels of *CCNB1, CCNB2* and *CDC20* mRNA (*n* = 3 each). **E** and **F** 2 × 10^6^ MCF7 cells were injected in the 4th inguinal mammary fat pad of female SCID mice with continuous supplementation of estrogen. Once the tumors were palpable, the mice were treated with vehicle (Captisol; *n* = 7), (*R*)-**9b** at 24 mg/Kg subcutaneously (*n* = 8), or orally with (*R*)-**9b** at 36 mg/Kg (*n* = 8), five times a week, for 4 weeks. The tumors were harvested, their weights were recorded and photographed. **G** Tumor lysates were subjected to immunoblotting using pACK1, CCNB1, CCNB2, CDC20 and Actin antibodies (*n* = 3 each). **H** RNA was prepared from the tumors, followed by qRT-PCR to determine the levels of *CCNB1, CCNB2* and *CDC20* mRNA (*n* = 3 each). Data are represented as the mean ± SEM. *p < 0.05; **p < 0.01; ***p < 0.001. For (**C**) and (**D**), unpaired two-tailed Student’s *t* test, and for (**E**) and (**H**), one way ANOVA was performed.

In order to assess the in vivo implications of ACK1 inhibition in TNBCs, Cal-148 xenografts were established in mammary fat pad of female SCID mice. Once the tumors were palpable, the mice were injected subcutaneously with 24 mg/Kg of (*R*)-**9b** for 4 weeks and the tumor volumes were measured. On reaching the endpoint, the mice were sacrificed and the tumors were harvested, weighed and photographed. (*R*)-**9b** exerted a significant suppression of TNBC xenograft tumor growth (Fig. 6A–C). RNA was prepared from the tumors, followed by qRT-PCR. A significant decrease in *CCNB1, CCNB2* and *CDC20* mRNA levels was observed upon (*R*)-**9b** treatment (Fig. 6D).

In addition, effect of (*R*)-**9b** was assessed in ER^+^/PR^+^ breast cancer xenografts. Briefly, MCF7 cells were implanted in mammary fat pad of female SCID mice. Once the tumors were palpable, the mice were treated orally (36 mg/Kg) or injected subcutaneously with 24 mg/Kg of (*R*)-**9b** for 4 weeks. The (*R*)-**9b** treated mice exhibited significant loss of tumor growth (Fig. 6E, F). To determine whether loss of tumor growth is due to compromised expression of cell cycle genes, xenograft tumor lysates were subjected to immunoblotting; (*R*)-**9b** treatment caused a marked decrease in CDC20, CCNB1 and CCNB2 protein levels (Fig. 6G). Further, RNA was isolated from the xenograft tumors and *CDC20, CCNB1* and *CCNB2* mRNA levels were examined. The (*R*)-**9b** treated breast xenograft tumors exhibited a significant reduction in *CDC20, CCNB1* and *CCNB2* mRNA levels, compared with vehicle treated mice (Fig. 6H). Together these data indicate that ACK1 inhibition could not only compromise ER^+^/PR^+^ and TNBC xenograft tumor growth by suppressing expression of cell cycle regulatory genes, but also reveal that the oral inoculation of compound is a viable drug delivery option.

### ACK1 inhibitor (*R*)-9b overcomes palbociclib-resistant breast tumor growth

**Fig. 7:**
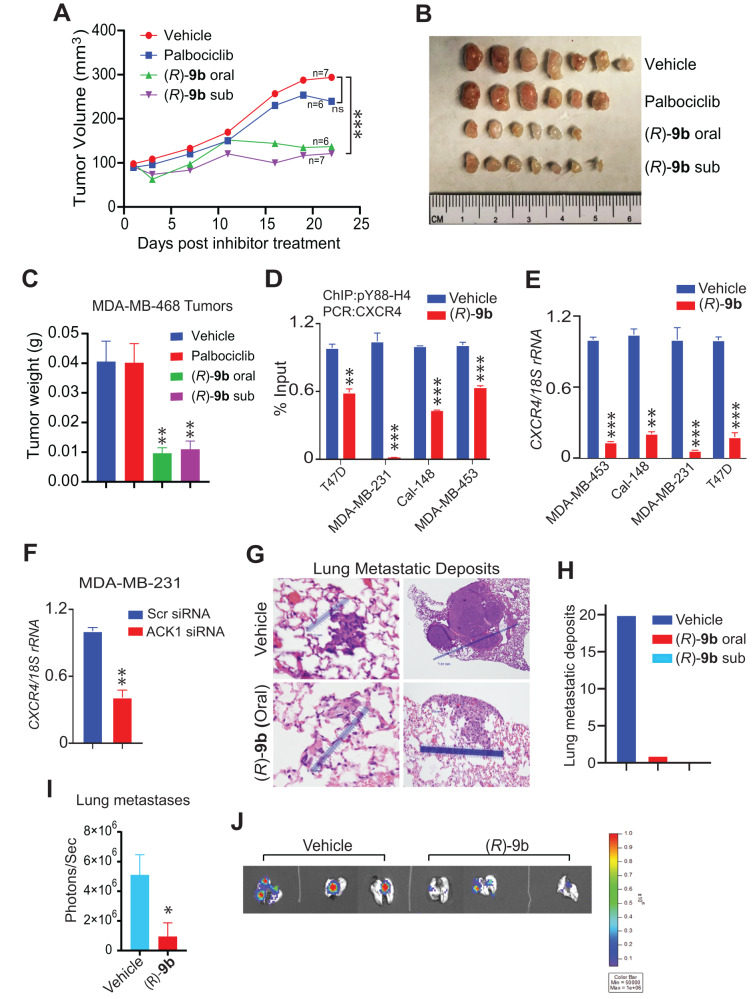
ACK1 inhibitor overcomes palbociclib-resistant breast cancer growth and metastasis. **A** 3 × 10^6^ MDA-MB-468 cells were injected in the 4th inguinal mammary fat pad of female SCID mice. Once the tumors were palpable, the mice were treated with either vehicle (Captisol; *n* = 7), Palbociclib at 80 mg/kg (*n* = 6), or (*R*)-**9b** at 36 mg/Kg (*n* = 6) orally. In addition, mice were injected with (*R*)-**9b** at 24 mg/Kg (*n* = 7) subcutaneously. Mice were treated five times a week, for 22 days. **B**, **C** The tumors were harvested and photographed, and the weights were recorded. **D** Vehicle or (*R*)-**9b** treated breast cancer cells were subjected to ChIP using pY88-H4 antibody, followed by qPCR for *CXCR4* gene. **E** *CXCR4* mRNA transcript levels in vehicle or (*R*)-**9b** treated MDA-MB-231 cells. *18S rRNA* was used as housekeeping gene. **F** MDA-MB-231 cells were transfected with *ACK1* or scrambled siRNA, followed by qRT-PCR to determine *CXCR4* mRNA transcript levels. **G** Representative lung metastatic deposits of MDA-MB-231 cells injected via the tail veins in female SCID mice, treated with either vehicle or 24 mg/Kg (*R*)-**9b** orally. **H** Graphical quantitation of lung metastatic deposit. **I** and **J** 5 × 10^5^ luciferase expressing 4T1 cells were injected into 4th inguinal mammary fat pad of female BALB/c mice. Once the tumors were palpable, mice were treated with either vehicle or (*R*)-**9b** at 24 mg/Kg (*n* = 3, each) subcutaneously for 2 weeks, five times a week. The metastases were assessed using IVIS. Data are represented as the mean ± SEM. *p < 0.05; **p < 0.01; ***p < 0.001. For (**A**, **C**–**F**, **I**), *p* values were calculated using unpaired two-tailed Student’s *t* test.

BT549 and MDA-MB-468 cell lines resistant to CDK4/6 inhibitors were observed to be sensitive to (*R*)-**9b** treatment (Fig. 5G, H). To validate these findings in breast tumors, MDA-MB-468 cells were injected in female SCID mice. Once palpable tumors were formed (5 weeks), mice were treated with vehicle (6% captisol), palbociclib (80 mg/Kg of body weight), (*R*)-**9b** (36 mg/Kg of body weight) by oral route, or (*R*)-**9b** (24 mg/Kg of body weight) by subcutaneous injection, five times a week. Formation of tumor was monitored for 3 weeks, vehicle and palbociclib**-**treated mice formed robust tumor growth, in contrast, (*R*)-**9b** treated mice, both oral and subcutaneous route, exhibited compromised tumors growth (Fig. 7A). At the end of the experiment, mice were humanely euthanized and tumors were excised (Fig. 7B), and weighed (Fig. 7C), exhibiting significant reduction in tumor weights. Weights of the mice were also monitored during this experiment, which showed modest decrease in weights in mice injected with palbociclib, in contrast, mice that were injected with (*R*)-**9b** did not exhibit significant loss of weight (Supplementary Fig. S6C). To further assess any pathological side effects of (*R*)-9b treatment, heart, spleen, kidneys, and livers from the mice were excised and stained with H&E. The organs from vehicle, (*R*)-**9b** (both, oral and subcutaneous) and palbociclib-treated mice did not reveal histological abnormalities (Supplementary Fig. S7A). Overall, these data suggest that breast tumors resistant to CDK4/6 inhibitors can be rendered susceptible to ACK1 inhibitor, (*R*)-**9b**.

### (*R*)-9b suppressed CXCR4 and impaired breast cancer metastasis

In addition to cell cycle regulatory genes, *CDC20*, *CCNB1* and *CCNB2*, *CXCR4* was another gene that was found to be significantly modulated upon (*R*)-**9b** treatment in RNA-sequencing data set (Supplementary Fig. S6D). Recent studies have reported the importance of *CXCR4* in breast cancer desmoplasia, metastasis and poorer clinical outcome [48–51]. We observed that ACK1 deposited pY88-H4 epigenetic marks in *CXCR4* promoter region (Supplementary Fig. S6E). Further, its suppression using (*R*)-**9b** not only erased these marks, but also inhibited *CXCR4* transcription (Fig. 7D, E). Further, genetic ablation of ACK1 by siRNA too suppressed transcription of *CXCR4* (Fig. 7F).

To validate the functional significance in vivo, we performed the tail vein assay of cancer metastasis. Briefly, MDA-MB-231 cells were injected in tail veins of female SCID mice and were treated with vehicle or 24 mg/Kg (*R*)-**9b** either subcutaneously or orally, for 5 days a week. The mice were euthanized and the lungs were harvested, preserved in 10% neutral buffered formalin, followed by H&E staining. Sections were examined by pathologist (C.W), and scored for metastatic nodules/deposits in 12 × 10 mm area of lung tissue. The vehicle treated group exhibited about 20 deposits ranging in size from 0.11–1.81 mm in a 12 × 10 mm area of lung tissue, in contrast, for the (*R*)-**9b** (oral) treated group, 2 deposits (0.44 mm deposit in a 14 × 12 mm area and 0.15 mm deposit in 15 × 14 mm area) were observed (Fig. 7G, H). Further, (*R*)-**9b** (subcutaneous) treated group exhibited no deposits (Fig. 7G, H).

In addition, we performed fluorescent imaging on mice orthotopically injected with luciferase expressing 4T1 cells (5 × 10^5^). Two weeks after inoculation into the mammary fat pads, mice were subcutaneously injected with vehicle (6% Captisol) or (*R*)-**9b** (24 mg/kg) five times a week for 2 weeks. Mice were then administered an intraperitoneal injection of D-luciferin, lungs were excised and imaged for metastases. A significant decrease in lung luminescence (photons/sec) was observed upon (*R*)-**9b** treatment (Fig. 7I, J). The lungs were also H&E stained, revealing distinct tumor nodules in vehicle treated mice (Supplementary Fig. S7B-i), in contrast, the nodules were absent in (*R*)-**9b** treated mice (Supplementary Fig. S7B-iii), suggesting the inhibitor could compromise breast cancer metastasis to lungs.

## Discussion

Breast cancer cells are known to harbor diverse molecular alterations and possess varied molecular landscapes, and thus discovering targetable molecules remains a challenge. In this report, we identified activated ACK1 as an oncoprotein that is activated in ER^+^, ER^+^/PR^+^, HER2^+^ and TNBC subsets of breast cancers. Two distinct mechanisms could potentially lead to ACK1 activation; (1) gene-amplification (in about 30% of breast cancers), and (2) activation by RTKs such as HER2, EGFR, MERTK, PDGFR and insulin receptor [27, 34]. These data opened doors for a new therapeutic modality for multiple subsets of breast cancer, inhibition of ACK1 using a potent small molecular inhibitor (*R*)-**9b**. The transcripts affected by loss of ACK1 activity belonged to the cell cycle regulating genes, epigenetically regulated by depositing the pY88-H4 marks. Consistent with that, inhibition of ACK1 using (*R*)-**9b** not only erased pY88-H4 epigenetic marks from *CCNB1*, *CCNB2* and *CDC20* genes, curbing the transcription, but also caused a cell cycle arrest in the G2/M phase. To our knowledge this is the first report that demonstrates the epigenetic control of cell cycle regulating genes by histone phosphorylation, which can be directly correlated with the hyper-functioning of ACK1 kinase activity in breast cancer.

Resistance to CDK4/6 inhibitors in clinical treatment is an unescapable problem. The molecular mechanisms responsible for resistance to CDK4/6 inhibitors appear to be diverse and likely to involve more than one signaling event. Several bypass signaling pathway mechanisms on CDK4/6 inhibitor adaption have been reported [18–20]. Cyclin B1 coded by the gene *CCNB1*, in association with CDK1 is crucial for regulating the transition of the G2/M phase of the cell cycle, orchestrating spindle checkpoint signaling [41]. A number of studies have implicated the tumor-promoting role of *CCNB1* in breast, cervical, lung, esophageal cancer and melanoma [52]. *CCNB1* has been suggested as a prognostic biomarker for ER^+^ breast cancer, indicating its role in hormone therapy resistance, signifying development of Cyclin B1 targeted therapy [53]. Similar to Cyclin B1, Cyclin B2 too is essential for G2/M (mitosis) transition and shown to be over-expressed in breast cancer [44], correlating with unfavorable prognosis and poor overall survival rates [44, 54]. Segregation of chromosome and the mitotic exit are initiated by the APC/C (anaphase-promoting complex/cyclosome) and its coactivator CDC20 [55]. CDC20 is overexpressed in breast cancer and exert a tumor-promoting role [56–58]. Simultaneous downregulation of these three crucial genes by ACK1 inhibitor resulted in G2/M arrest in four different subtypes of breast cancer, thus uncovering a new therapeutic vulnerability for CDK4/6-inhibitor resistant cells, which primarily developed insensitivity to palbociclib by continuing with G1 to S cell cycle transition.

Induction of ER signaling with estrogen induces SIAH2, which in turn causes proteasomal degradation of ACK1 [59, 60]. ER^+^ breast cancer samples could potentially have lower staining of ACK1 due to estrogen/ER/SIAH signaling. Interestingly, the luminal type, ER^+^ and PR^+^ tumors exhibit high pACK1 levels (Fig. 1A), opening a possibility that luminal tumors could compensate negative effects of estrogen on ACK1 protein levels by increased ACK1 activation.

The histone deacetylases HDAC1/HDAC2 sustain the phosphorylation of the checkpoint kinases ATM, CHK1 and CHK2, activity of the cell cycle gatekeeper kinases WEE1 and CDK1, and induction of the tumor suppressor p53 in response to stalled DNA replication [61]. We assessed effect of ACK1 inhibition; poor γH2AX staining upon (*R*)-**9b** treatment suggest that this compound does not cause DNA damage, especially double strand breaks (Supplementary Fig. S8). Interestingly, CDK1 and 2, and WEE1 expression is affected by (*R*)-**9b** treatment in MCF-7 and MDA-MB-453, but not in SKBR3 or normal MCF10A (Supplementary Fig. S9). Precisely how expression of CDKs and WEE1 is affected by ACK1 inhibition is not entirely clear, however, this could also contribute toward cell cycle arrest upon (*R*)-**9b** treatment.

Spindle poisons such as paclitaxel have extensively used in breast cancer treatment. Paclitaxel prevents cell division by promoting the assembly of stable microtubules especially β-tubulin heterodimers and inhibits their depolymerization, accordingly, exposed cells are arrested in the G2/M-phase of the cell cycle. (*R*)-**9b** too causes G2/M arrest, but by distinct epigenetic mechanism. Peripheral neuropathy is a major dose-limiting side effect of paclitaxel [62], thus, it is plausible to combine (*R*)-**9b** with the lower concentration of paclitaxel to achieve efficacy without the side effects.

(*R*)-**9b** hits both palbociclib-resistant and palbociclib-sensitive cell lines. For example, BT549 and MDA-MB-468 are Rb-negative and palbociclib-resistant, however, these two lines were sensitive to (*R*)-**9b** treatment (Fig. 5G–J). Further, MCF7, SKBR, and HCC-1395 are Rb-positive and palbociclib-sensitive, were also sensitized by (*R*)-**9b**. Recently, we have shown that ACK1 regulates AR levels, and AR^+^ prostate cancer cells are quite sensitive to (*R*)-**9b** [21, 22]. AR is expressed in more than 70% of primary breast cancer and its expression is correlated to ER. AR prevalence is higher in ER^+^ early breast tumors than ER^−^ tumors (74.8% vs. 31.8% of cases, respectively) [63]. Patients with ER^−^/AR^+^ tumors have better outcomes [64], accordingly, a phase II clinical trial evaluating AR-antagonist, enzalutamide in AR^+^ TNBC showed a clinical benefit rate of 33% at 16 weeks, and a median progression-free survival duration of 3.3 months [65, 66]. Interestingly, a phase I/II clinical trial of another AR antagonist, an androgen synthesis inhibitor, abiraterone in women with advanced ER^+^/AR^+^ breast cancer presented a clinical benefit rate of 20% in 24 weeks with a median progression-free survival duration of 2.8 months (NCT00755885). Thus, (*R*)-**9b** could have pantropic effect in breast cancers because of not only its ability to suppress cell cycle genes, but also AR expression. In addition, we have recently shown immune modulatory properties of (*R*)-**9b** [38], which could also contribute to the pantropic effect of (*R*)-**9b**. Considering that ACK1 inhibition has been shown to be quite effective in suppressing prostate tumor growth [21, 22], pantropic effect of (*R*)-**9b** could be evident in both of these hormonally regulated tumors.

*CXCR4* gene is implicated in BC metastasis that not only induces desmoplasia- confer immune resistance of the primary tumor, but its inhibition sensitizes the tumor cells to immune infiltration and regression [48–50]. The CXCR4-LASP1 axis regulates stability of nuclear localized Snail1 and likely target to overcome breast cancer metastasis [50, 67]. (*R*)-**9b** not only reversed *CXCR4* expression, but also circumvented BC metastasis to lungs, underscoring the physiological relevance ACK1 inhibition in later stages of disease. Taken together with tumor inhibitory data, our studies intensify the necessity of exploring ACK1 as a prospective target in all subsets of breast cancer.

## Materials and methods

### Cell lines

MDA-MB-231, MDA-MB-453, MDA-MB-468, BT549, HCC-1395, T47D and MCF7 cells were obtained from ATCC. Cal-148 cells were obtained from Leibniz Institute DSMZ-German Collection of Microorganisms and Cell Cultures. MDA-MB-231 and MDA-MB-453 were grown in DMEM supplemented with 10% FBS (Invitrogen). MCF7 cells were grown in MEM with 10% FBS (Invitrogen). Cal-148 cells were grown in DMEM with 10% FBS (Invitrogen) and human EGF (10 ug/ml). HCC-1395, SKBR3, and T47D were grown in RPMI supplemented with 10% FBS. All cultures were maintained with 50 units/ml of penicillin/streptomycin (Invitrogen) and cultured in 5% CO_2_ incubator. Mycoplasma testing was performed every 2 months and cell line authentication was performed by STR profiling.

### Proliferation assay, apoptosis assay and cell cycle analysis

For proliferation assay, 5 × 10^4^ cells were plated in 6-well plate and treated with either vehicle (DMSO) or varying concentrations of (*R*)-**9b** or palbociclib in complete media for 96 h and number of viable cells were counted by trypan blue exclusion assay. Breast cancer cell lines were treated with vehicle or varying concentrations of (*R*)-**9b** for 72 h. The cells were harvested, washed 2 times with PBS and incubated with 3 ul of Annexin V-FITC antibody according to the manufacturers protocol (BD Biosciences). Five µl Propidium iodide was used to stain necrotic cells. The cells were acquired using the BD FACSCalibur machine (BD Biosciences) and data was analyzed using FlowJo software. Cell cycle analysis was performed as mentioned earlier [24]. Briefly, vehicle or (*R*)-**9b** (48 h) treated cells were harvested by trypsinization, washed 2 times with PBS, and fixed using chilled 70% Ethanol for 1 h, followed by 2 washes with PBS. The cell pellets were treated with 50 µl RNase A (100 µg/ml; Sigma) for 15 min at 37 °C and Propidium Iodide (PI; 50 µg/ml, Sigma) was added to these cells, followed by acquisition using BD FACS Canto-II or FACSCalibur machines. The data were analyzed using FlowJo software.

### Immunoprecipitation and western blot analysis

Vehicle or (*R*)-**9b** (5 µM, overnight in serum free media) treated cells were lysed by sonication in receptor lysis buffer (RLB) [27] and lysates were fractionated by SDS-PAGE, and transferred onto a PVDF membrane (Immobilon). The blocked membranes (5% milk or 3% BSA, 1 h, room temperature) were incubated with the following primary antibodies: ACK1 mouse monoclonal antibody (1:1000; Santacruz Biotechnology), pACK1 mouse monoclonal antibody (1:1000 EMD Millipore), Actin mouse monoclonal antibody (1:10,000; Sigma), histone H4 mouse monoclonal antibody (1:3000; Cell Signaling Technology), Cyclin B1 mouse monoclonal antibody (1:1000; Santacruz Biotechnology), Cyclin B2 mouse monoclonal antibody (1:1000; Santacruz Biotechnology), CDC20 rabbit monoclonal antibody (1:1000; Cell Signaling Technology). The blots were washed and the signals visualized by enhanced chemiluminescence (ECL) system according to manufacturer’s instructions (GE Healthcare). For detection of pY88-H4 and pY284-ACK1, cell lysates were immunoprecipitated using 3–4 µg of respective phospho-specific antibody coupled with protein A/G-sepharose beads (Santacruz Biotechnology), followed by immunoblotting using H4 or ACK1 antibody respectively.

### RNA sequencing

MDA-MB-453 and HCC-1395 cells were treated with either vehicle or (*R*)-**9b** (2.5 µM, overnight), cells were harvested and the RNA was processed followed by sequencing. The raw files were analyzed using Partek Flow analyzer.

### Chromatin immunoprecipitation (ChIP) and ChIP-sequencing

Cell pellets were resuspended in RLB buffer and sonicated and the soluble chromatin was incubated with antibodies and Protein-G/A magnetic beads. The complexes were washed with RLB buffer, followed by ChIP buffer 1 and 2 (Active Motif), eluted with elution buffer and subjected to Proteinase-K treatment. ChIP DNA was purified using PCR DNA purification columns (Qiagen). For ChIP-Seq, MDA-MB-453 cells (5 × 10^7^ cells) were treated with vehicle or (*R*)-**9b**. Cell pellets were resuspended in RLB buffer and sonicated for 25 s. The soluble chromatin was incubated overnight at 4 °C with antibodies and protein-G/A magnetic beads [21]. Ten nanograms of immunoprecipitated DNA was used to generate sequencing libraries using the Kapa Hyper Prep Kit (Roche Sequencing Solutions Inc., Pleasanton, CA). The size and quality of the library was evaluated using the Agilent BioAnalyzer (Agilent Technologies, Inc., Santa Clara, CA), and the library was quantitated with the Kapa Library Quantification Kit. Each enriched DNA library was then sequenced on an Illumina NextSeq 500 sequencer to generate 40–50 million 75-base paired-end reads (Illumina, Inc., San Diego, CA). The raw sequence data were aligned using BowTie 2 [68], and the binding sites were identified using the MACS peak-finding software [69].

### Quantitative RT-PCR and ChIP-qPCR

Real-time quantitative PCR analyses were performed using the ABI PRISM 7900HT Sequence Detection System (Applied Biosystems) as described earlier [70]. Briefly, the PCR was carried out with SYBR Green PCR Master Mix (Applied Biosystems) using 2 µl of cDNA (or ChIP DNA) and the primers in a 20 µl final reaction mixture. Dissociation curves were generated for each plate to verify the integrity of the primers. Data were analyzed using SDS software version 2.2.2 and exported into an Excel spreadsheet. The 18S or Actin data were used for normalizing the gene values. The sequences for the primers are in Supplementary Table 12.

### Mouse xenograft studies

All animal experiments were performed using the standards for humane care in accordance with the NIH Guide for the Care and Use of Laboratory Animals. Mice were obtained from Charles River Laboratories and experiments were performed according to IACUC protocols (IACUC # 20180259) approved in writing by Washington University in St. Louis Department of Comparative Medicine (DCM). 1.5 × 10^6^ Cal-148 or MCF7, or 3 × 10^6^ MDA-MB-468 cells were suspended in 200 µl of PBS with 50% matrigel (BD Biosciences) and were implanted subcutaneously into the mammary fat pad (between fourth and fifth inguinal) of female SCID C.B17 mice (*n* = 8 per group). Once the tumors were palpable (about 4–5 weeks), mice were randomly chosen for subcutaneous injection (Cal-148 and MCF7; 24 mg/Kg body weight) or given oral gavage with (*R*)-**9b** (MCF7; 36 mg/Kg body weight) resuspended in 6% Captisol (or 6% Captisol in PBS as vehicle), five times a week, for 4–5 weeks. Tumor volumes were measured twice a week using calipers. At the end of the study, all mice were humanely euthanized, tumors extracted and weighed.

### Tail vein metastasis studies

MDA-MB-231 cells (2 × 10^5^ in PBS), were injected intravenously in the lateral tail vein of female SCID mice. After 10 days, the mice were randomly chosen for treatment with either vehicle, (*R*)-**9b** (36 mg/Kg body weight) orally or subcutaneously (s.c.) (24 mg/Kg body weight) for 5 days a week, for 2 weeks. The mice were sacrificed and the lungs were harvested and fixed in 10% neutral buffered formalin. The number of metastatic foci per lung were scored in a blinded fashion by a pathologist (C.W.).

### Orthotopic lung metastasis study

Female Balb/c mice were orthotopically injected with luciferase expressing 4T1 cells (5 × 10^5^). Two weeks after inoculation into the 5th mammary fat pads, mice were subcutaneously injected with vehicle (6% captisol) or (*R*)-**9b** (24 mg/Kg) five times a week, for 2 weeks. Mice were then intraperitoneally injected with D-luciferin, humanely euthanized, lungs were excised and assessed for metastases using both fluorescent in vivo imaging system and H&E staining.

### Breast cancer tissue microarray (TMA) and human subjects

Dr. J.C.R. at the School of Medicine in Pontificia Universidad Católica de Chile in Santiago, Chile has assembled a set of breast cancer TMAs comprising about 400 de-identified breast cancer tumor cores. The TMAs were stained with pY284-ACK1 and ACK1 antibodies. The breast TMA used in this study is exempt from IRB approval, as no personal information about the patients was sought.

### Immunocytochemistry

Breast cancer cells were grown over coverslips and treated with vehicle or (*R*)-**9b**. The cells were fixed in 2% paraformaldehyde and permeabilized. The cells were stained with phospho-serine 139-γH2AX antibody (Red), phalloidin (green) and DAPI. Fluorescent microscopic images were captured.

### Immunohistochemical (IHC) staining

Antibodies for pY284-ACK1 (1:300; Sigma) and ACK1 (1:50; Santacruz Biotechnology) have been characterized for IHC staining of the TMAs [24, 25, 27]. The TMA slides (including positive and negative controls) were stained with pY284-ACK1 antibodies and ACK1 antibodies overnight. Negative controls were included by omitting pTyr284-ACK1 antibodies during primary antibody incubation or incubating pTyr284-ACK1 antibody with purified activated-ACK1 protein prior to TMA staining. The TMA staining was examined in a blinded fashion by two independent pathologists (C.O’C. and I.S.H). Results were scored into four grades according to the intensity of staining: 0 (no staining), 1 (mild staining), 2 (moderate staining) and 3 (strong staining). Any discrepancies were resolved in a consensus conference.

### Statistical analysis of TMA

Data were expressed as the mean ± SEM. Data between two groups were analyzed with unpaired Student’s *t* tests. Boxplots were used to summarize the intensity distribution at each progression stage. The Kruskal–Wallis test was performed to examine if there is an overall difference for pY284-ACK1 and ACK1 within each of the four subtypes of breast cancer. All analyses were conducted using Graphpad Prism (Graphpad Software Inc, California, USA) and R (version 4.1.1). All tests were two sided and statistical significance was defined at the 5% alpha level.

### Statistical analysis

For all the experiments, the sample size was chosen based on prior studies that showed significant effects with similar sample sizes. No data was excluded from the analysis. For mice tumor studies, mice were randomly assigned to two or more groups prior to the injection of cells or drug. Blinding was not done for any of the experiments, including the animal assignments for tumor formation studies and molecular analysis. Data were expressed as the mean ± SEM. Data between two groups were analyzed with unpaired Student’s *t* tests using Graphpad Prism (Graphpad Software Inc, California, USA). A *p value of ≤0.05 is considered statistically significant. IC_50_ calculation was done using a variable slope, four parameter, non-linear regression.

## Supplementary information

Supplementary Figures 1-9, & Tables 1-9 and 11

Supplementary Table 10

## Data Availability

The authors declare that all data that support the findings of this study are available within the paper and Supplementary files. The GEO accession is GSE203232.
