# Supplementary material for: Epigenetic reprogramming of cell cycle genes by ACK1 promotes breast cancer resistance to CDK4/6 inhibitor
Source: Oncogene. 2023 Jun 17;42(29):2263–77. doi: 10.1038/s41388-023-02747-x (PMC10348910; doi:10.1038/s41388-023-02747-x)
Supplement: Supplementary file 1 — Supplementary Figures 1-9, & Tables 1-9 and 11 [file 41388_2023_2747_MOESM1_ESM.pdf]

Supplementary Information for:

Epigenetic Reprogramming of Cell Cycle Genes by ACK1 Promotes Breast Cancer Resistance to CDK4/6 Inhibitor

Mithila Sawant[1,2#], Audrey Wilson[1,2#], Dhivya Sidaran[1,2], Kiran Mahajan[1,2,4], Christopher O'Conor[3], Ian S. Hagemann[3], Jingqin Luo[4], Cody Weimholt[3,4], Tiandao Li[5], Juan Carlos Roa[6], Akhilesh Pandey[7,8], Xinyan Wu[7,8], Nupam P. Mahajan[1,2,4,9]*

Supplementary Figure S1: ACK1 expression in metastatic breast cancer datasets from cBioPortal

Supplementary Figure S2: ACK1 inhibition using small molecular inhibitor (*R*)-9b leads to induction of apoptosis in Breast cancer cells

Supplementary Figure S3: ACK1 inhibition using (*R*)-9b leads to differential regulation of cell cycle genes in Triple negative breast cancer

Supplementary Figure S4: ACK1 epigenetically regulates cell cycle genes by deposition of pY88-H4 activating marks

Supplementary Figure S5: Bioinformatic analysis of pY88-H4 deposition in MDA-MB-453 cells

Supplementary Figure S6: ACK1 knockdown leads to G2/M arrest in breast cancer cells

Supplementary Figure S7: ACK1 inhibition does not cause toxicity in vital organs (A). ACK1 inhibition reduces lung metastatic tumor load in 4T1-luc cell injected mice (B).

Supplementary Figure S8: ACK1 inhibition using does not cause double-stranded DNA breaks in breast cancer cell lines

Supplementary Figure S9: Effect of ACK1 inhibition on CDKs and WEE1.

Supplementary Tables

Supplementary Table S1. Intensities of pY284-ACK1 and ACK1 in breast cancer TMA.

Supplementary Table S2. Kinase profiling Report of ACK1.

Supplementary Table S3: Permeability of (R)-9bMS in Caco-2 Assay

Supplementary Table S4: Cytochrome P450 (CYP) inhibition in human liver microsomes

Supplementary Table S5: (i) Protein binding results of (*R*)-9bMS and control compound in human plasma. (ii) Protein binding results of (*R*)-9bMS and control compound in rat plasma

Supplementary Table S6: (i) Stability of (*R*)-9b in Simulated Gastric Fluid (SGF). (ii) Stability of (R)-9b in Simulated Intestinal Fluid (SIF)

Supplementary Table S7: Pharmacological profiling of (*R*)-9b

Supplementary Table S8. Cell proliferation regression analysis of breast cell lines

Supplementary Table S9. Genes downregulated upon ACK1 inhibition.

Supplementary Table S10: ChIP sequencing analysis of vehicle- or (*R*)-9b-treated MDA-MB-435 cells immunoprecipitated (ChIP) with pY88-H4 antibody,

Supplementary Table S11: A. Gene Annotated Peaks in Vehicle and (*R*)-9b treated sample. **B.** Differentially modulated gene types in Vehicle and (*R*)-9b treated samples

Supplementary Table S12: List of Primers

Supplementary Figure S1

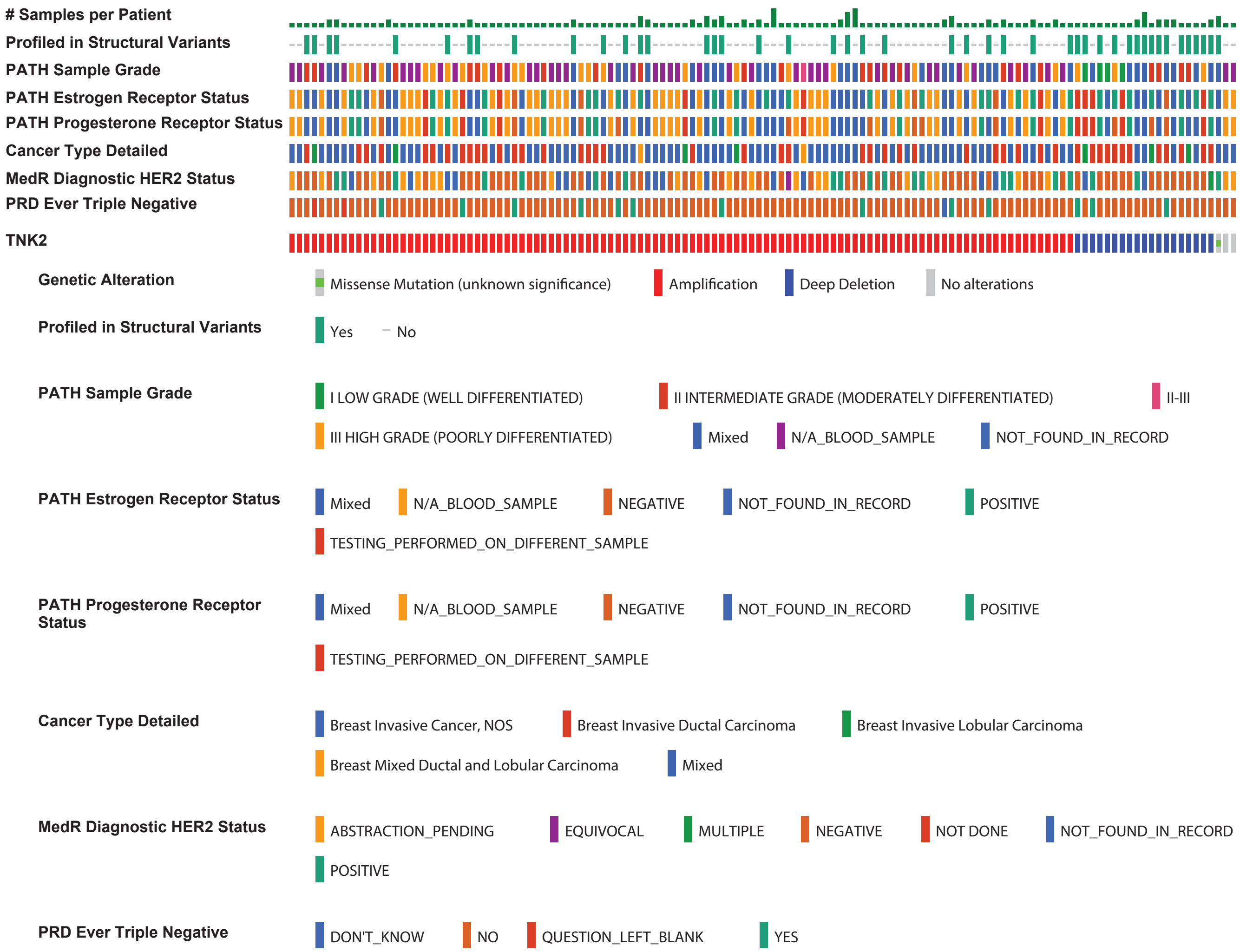

Supplementary Figure S1: ACK1 expression in metastatic breast cancer datasets from cBioPortal

Supplementary Figure S2

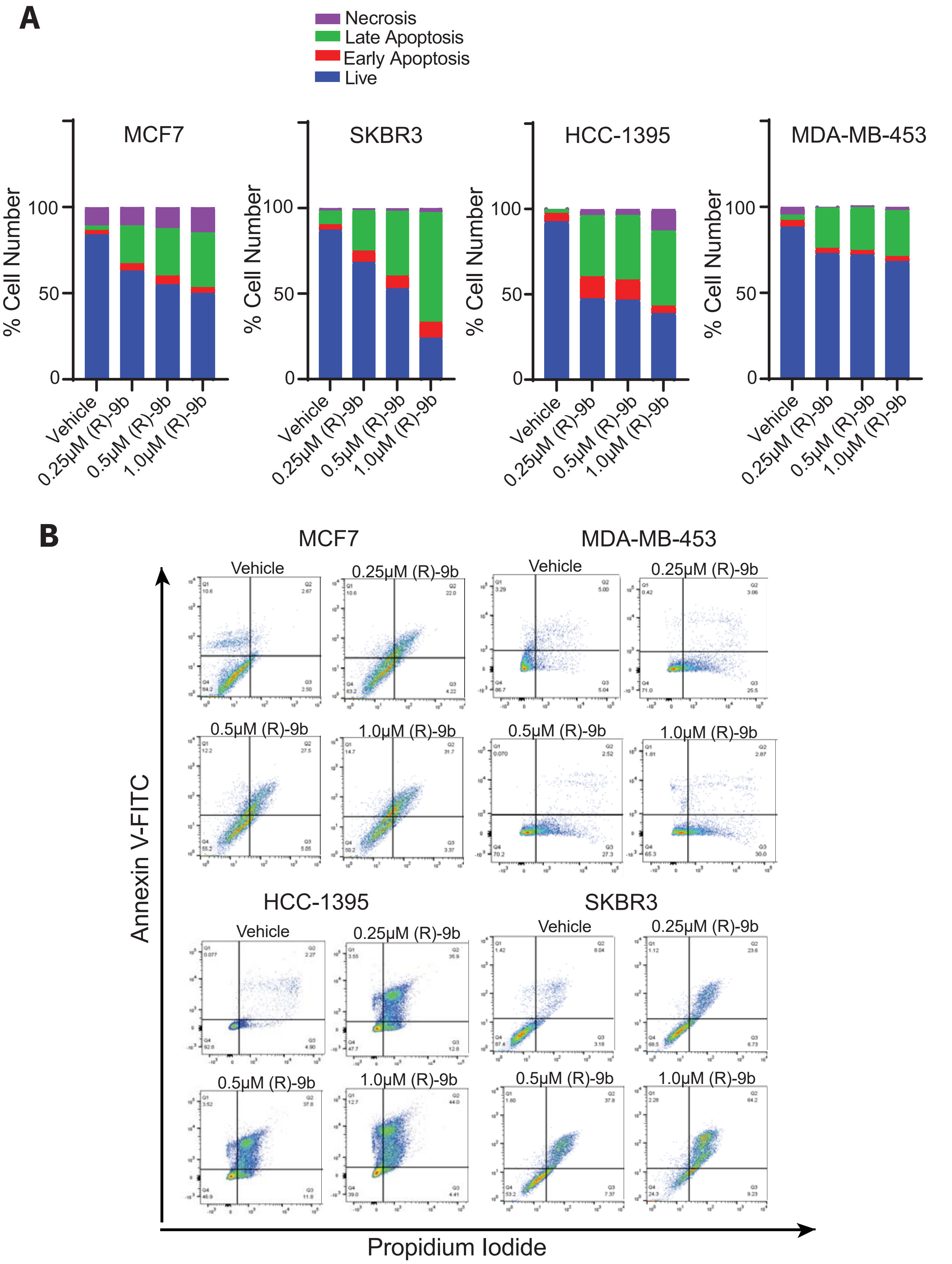

Supplementary Figure S2: ACK1 inhibition using small molecular inhibitor (*R*)-9b leads to induction of apoptosis in Breast cancer cells

(A) MCF7, SKBR3, HCC-1395 and MDA-MB-453 cells were treated with 0.25µM, 0.5µM and 1 µM (*R*)-**9b** for 96h. Cells were harvested and stained with Annexin-V FITC/PI according to the manufacturer's protocol. Quantification of the live, early apoptotic, late apoptotic and necrotic cells is represented using stacked columns.

(B) Representative scatter plots of cell cycle analysis are shown. (n=3 biologically independent experiments).

Supplementary Figure S3

A

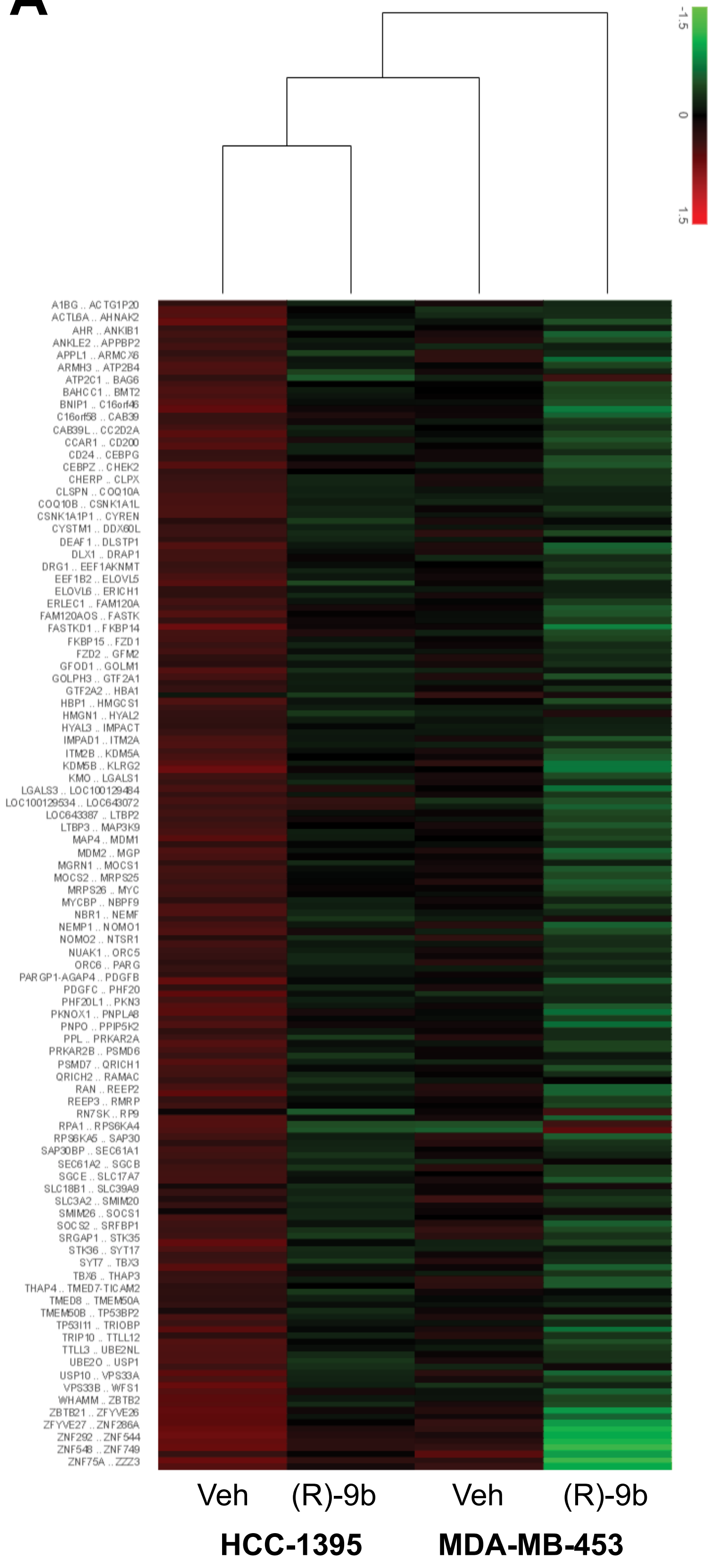

C

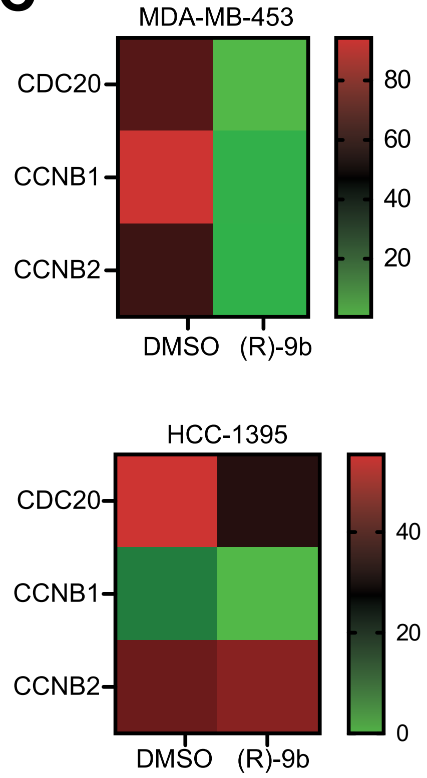

B

Cell Line	Gene set	Description	Enrichment score	P-value	Genes in list
HCC-1395	path:hsa04110	Cell cycle	7.3868	0.000619376	119
MDA-MB-453	path:hsa04110	Cell cycle	8.32013	0.000243564	117

Supplementary Figure S3: ACK1 inhibition using (*R*)-9b leads to differential regulation of cell cycle genes in Triple negative breast cancer

(A) Heat map representing differentially regulated genes on (*R*)-**9b** treatment in HCC-1395 and MDA-MB-453 RNA sequencing analysis.

(B) Pathway analysis of genes affected by (*R*)-**9b** treatment in HCC-1395 and MDA-MB-453 cells.

(C) Heat map representing cell cycle genes *CCNB1*, *CCNB2* and *CDC20* regulated by ACK1 inhibition using (*R*)-**9b** treatment in HCC-1395 and MDA-MB-453 cells.

Supplementary Figure S4

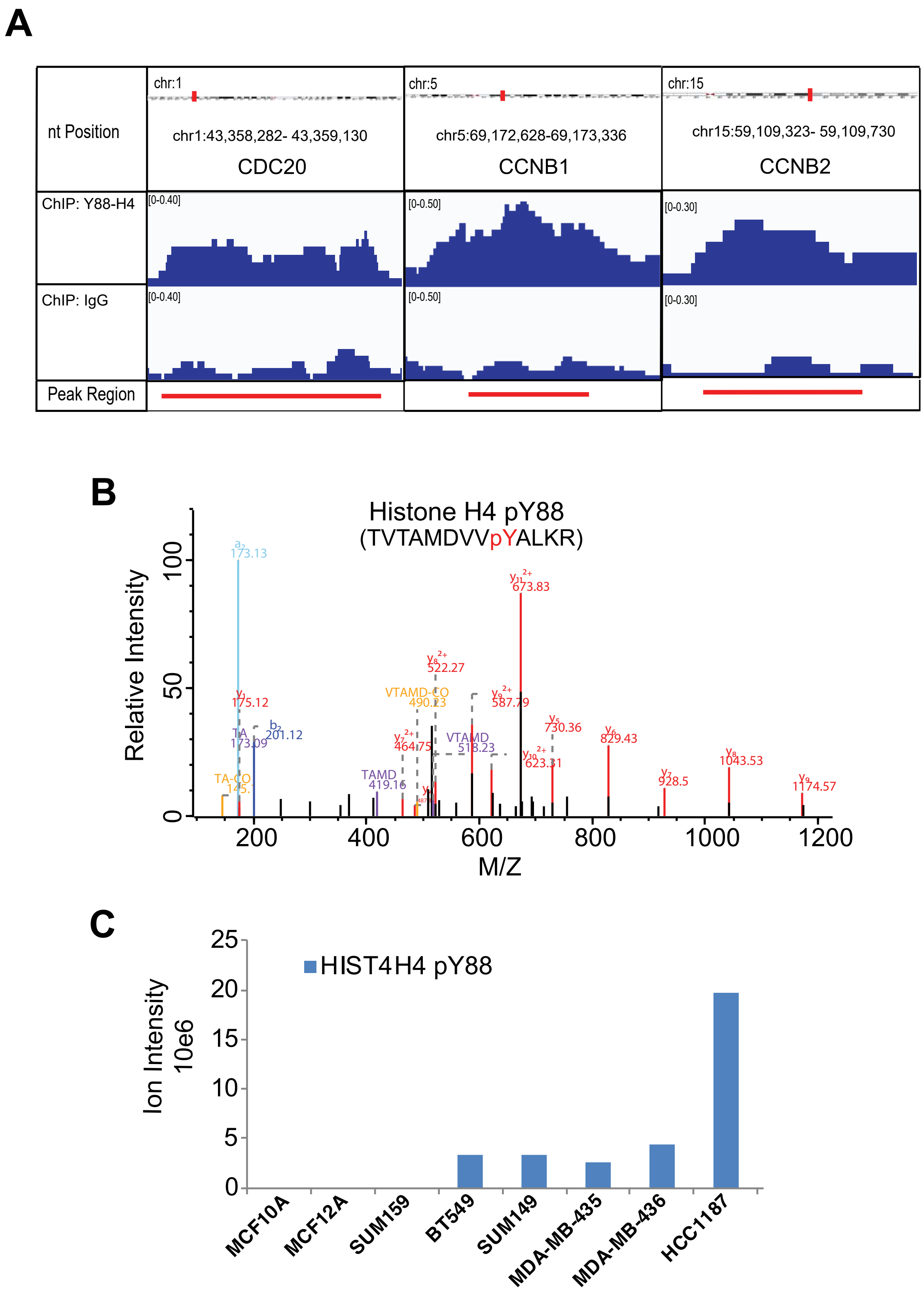

Supplementary Figure S4: ACK1 epigenetically regulates cell cycle genes by deposition of pY88-H4 activating marks

(A) pY88-H4 ChIP-sequencing in VCaP cells revealing peaks in *CDC20, CCNB1* and *CCNB2* genes. IgG is used as a negative control.

(B) Mass spectrometric detection of pY88-H4 expression in breast cancer cells.

(C) Mass spectrometric analysis of pY88-H4 in normal and breast cancer cell lines.

Supplementary Figure S5

A

Homer Known Motif Enrichment Results (MDA-MB-453_Veh_vs_INPUT_motif)

Rank	Motif	Name	P-value	log P-pvalue	q-value (Benjamini)	# Target Sequences with Motif	% of Targets Sequences with Motif	# Background Sequences with Motif	% of Background Sequences with Motif	Motif File	SVG
1	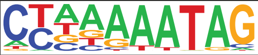	Mef2a(MADS)/HL1-Mef2a.biotin-ChIP-Seq(GSE21529)/Homer	1e-2	-5.405e+00	1.0000	99.0	10.59%	3972.8	8.12%	motif file (matrix)	svg
2	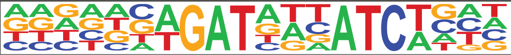	GATA(Zf),IR3/iTreg-Gata3-ChIP-Seq(GSE20898)/Homer	1e-2	-5.075e+00	1.0000	31.0	3.32%	992.5	2.03%	motif file (matrix)	svg
3	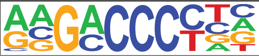	LRF(Zf)/Erythroblasts-ZBTB7A-ChIP-Seq(GSE74977)/Homer	1e-2	-4.784e+00	1.0000	115.0	12.30%	4819.1	9.85%	motif file (matrix)	svg

B

Gene Ontology (GO): Molecular functions

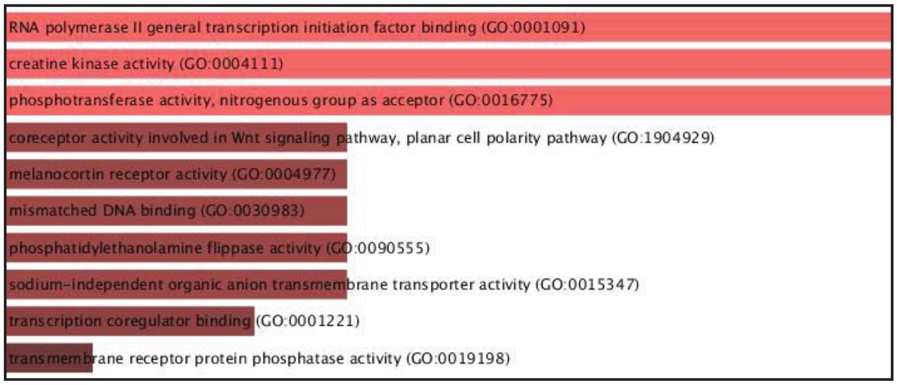

C

KEGG Pathway Analysis

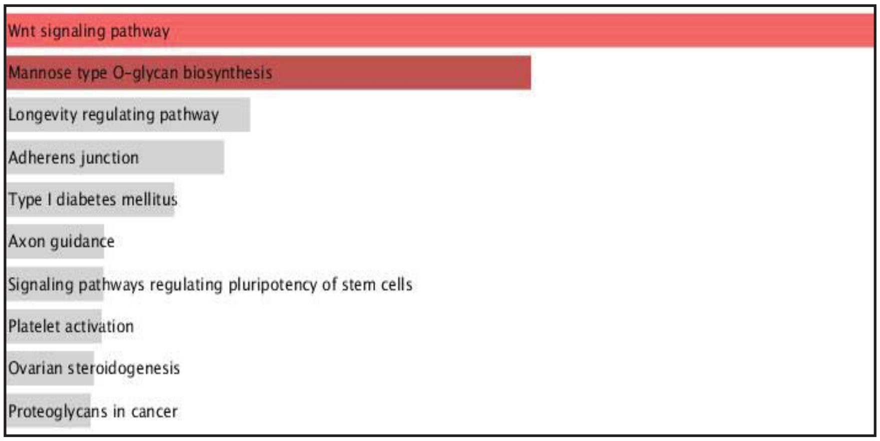

Supplementary Figure S5: Bioinformatic analysis of pY88-H4 deposition in MDA-MB-453 cells

(A) Homer known motif enrichment results for pY88-H4 ChIP in MDA-MB-453 cells.

(B) Molecular functions of pY88-H4 in MDA-MB-453 cells as assessed by Gene Ontology analysis.

(C) KEGG pathway analysis of genes regulated by pY88-H4 deposition in mDA-MB-453 cells.

Supplementary Figure S6

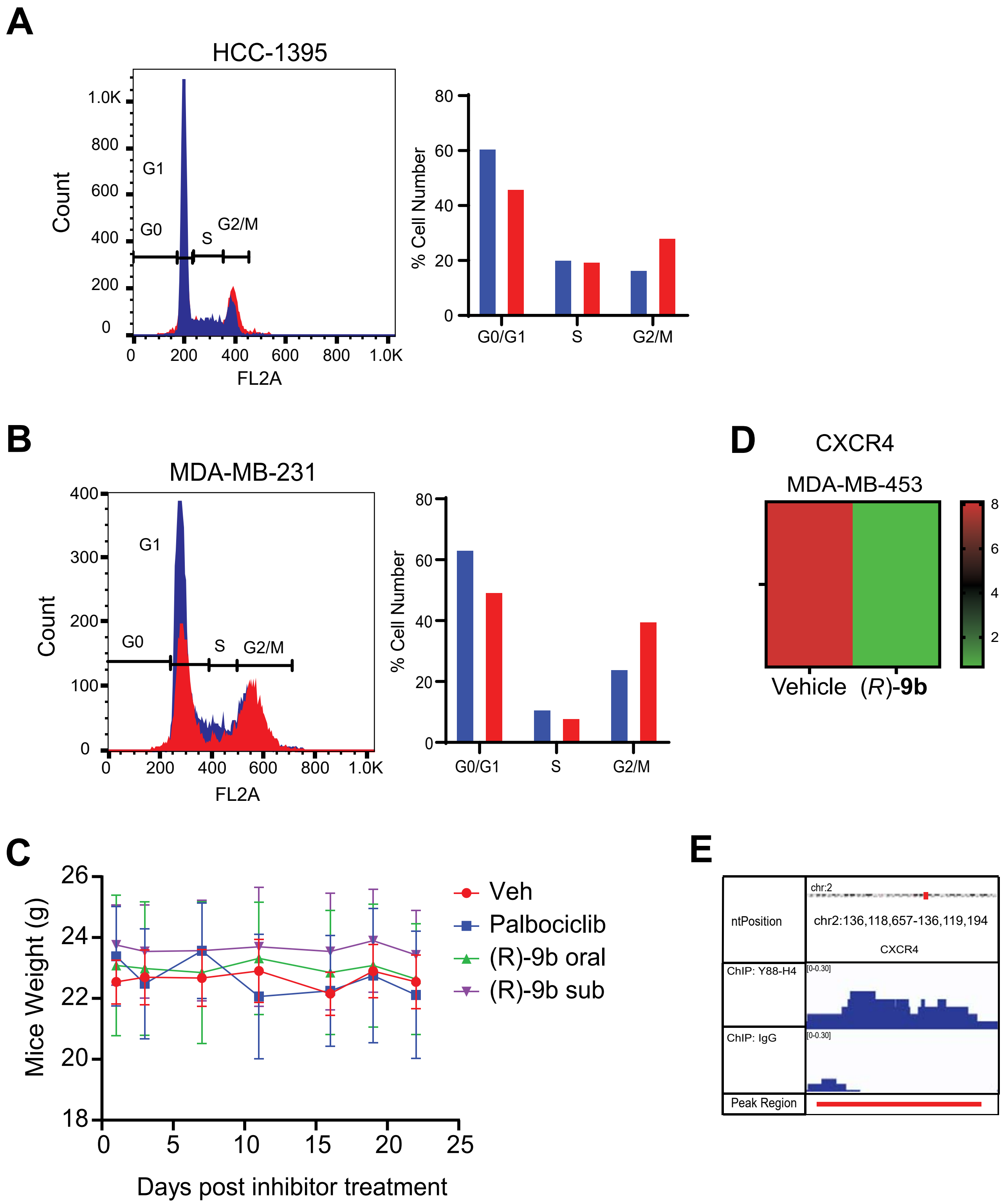

Supplementary Figure S6: ACK1 knockdown leads to G2/M arrest in breast cancer cells

(**A**) HCC-1395 and **(B)** MDA-MB-231 cells were transfected with scramble siRNA or ACK1 siRNA for 72h, and cell cycle arrest was studied using Propidium iodide staining. A representative histogram overlay of scramble siRNA control and ACK1 siRNA is shown with bar graphs representing quantitation of cells in different phases of cell cycle adjacent to it.

(**C**) Weights of the mice treated with vehicle, palbociclib, and (*R*)-**9b.**

(**D**) RNA sequencing analysis of *CXCR4* mRNA expression in vehicle and (*R*)-**9b** treated MDA-MB-453 cells.

(**E**) pY88-H4 ChIP-sequencing revealing peak in *CCXR4* gene. IgG is used as a negative control.

Supplementary Figure S7

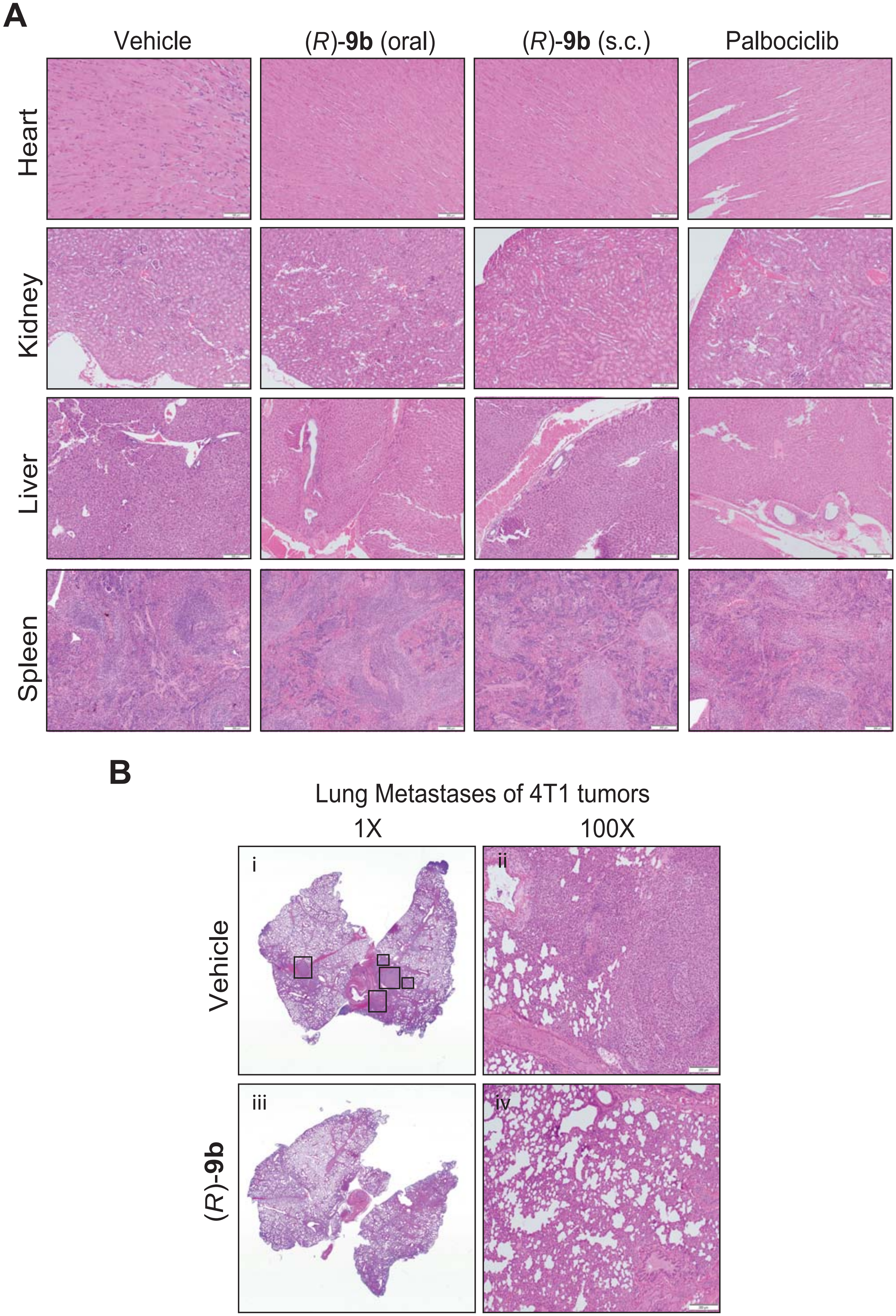

Supplementary Figure S7: ACK1 inhibition reduces lung metastatic tumor load in 4T1-luc injected mice without induction of vital organ toxicity

(A) H&E analysis of vital organs (heart, kidney, liver, and spleen) from vehicle, (*R*)-**9b** (oral or subcutaneous) and palbociclib treated mice injected with MDA-MB-468 cells.

(B) 4T1-luc injected mice were treated with either vehicle or (*R*)-**9b** for 2 weeks. After fluorescent imaging, lungs were excised, sectioned, H&E stained, and imaged to show tumor load. **(i & ii)** Vehicle treated mice, **(iii & iv)** (*R*)-**9b** treated mice.

Supplementary Figure S8

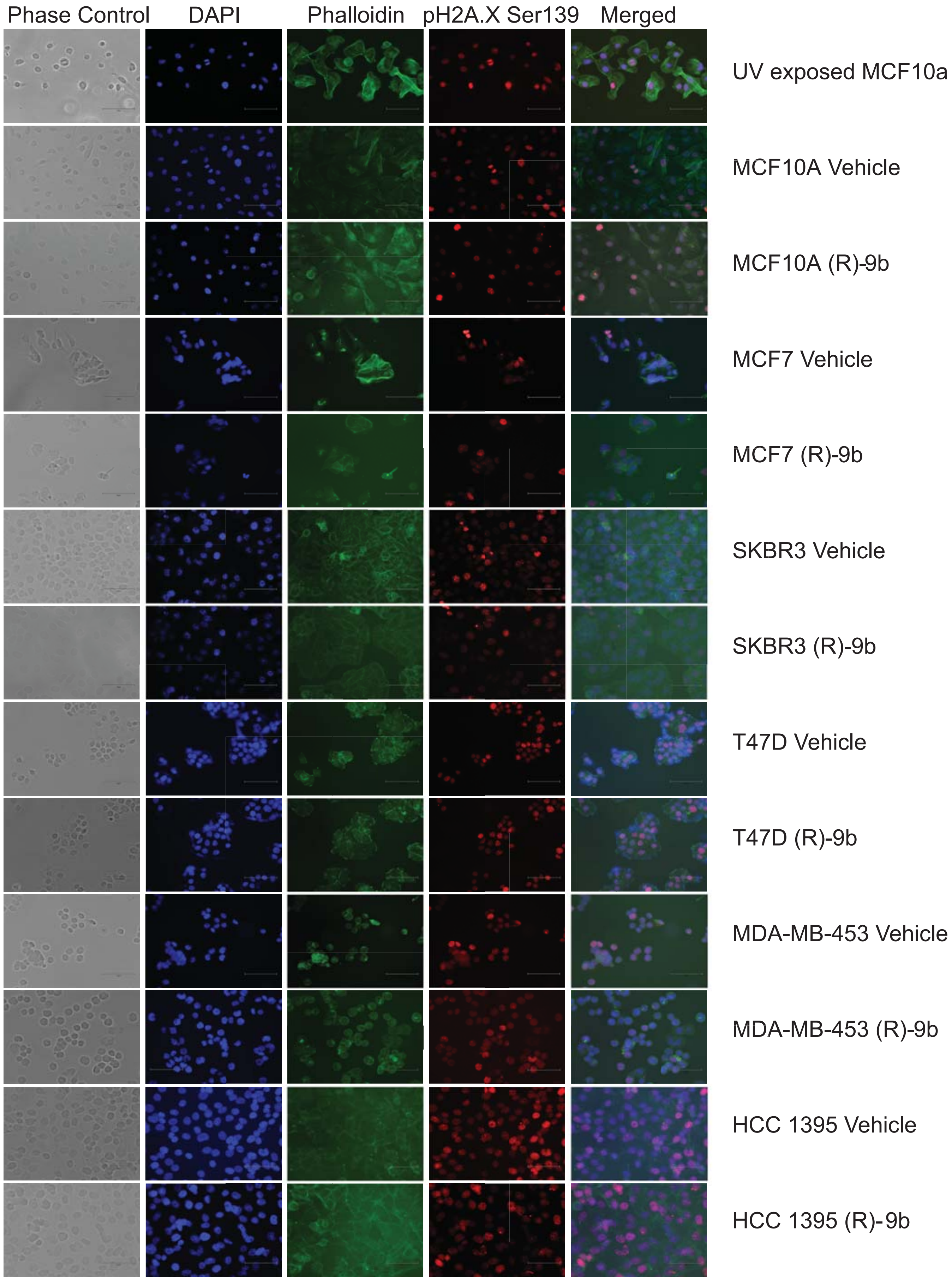

Supplementary Figure S8: ACK1 inhibition using does not cause double-stranded DNA breaks in breast cancer cell lines

Breast cancer cell lines were treated with either vehicle or (*R*)-**9b** for 48h and stained with phospho-serine 139-γH2AX antibody (Red), phalloidin (green) and DAPI. Representative images of fluorescent microscopic analysis are shown.

Supplementary Figure S9

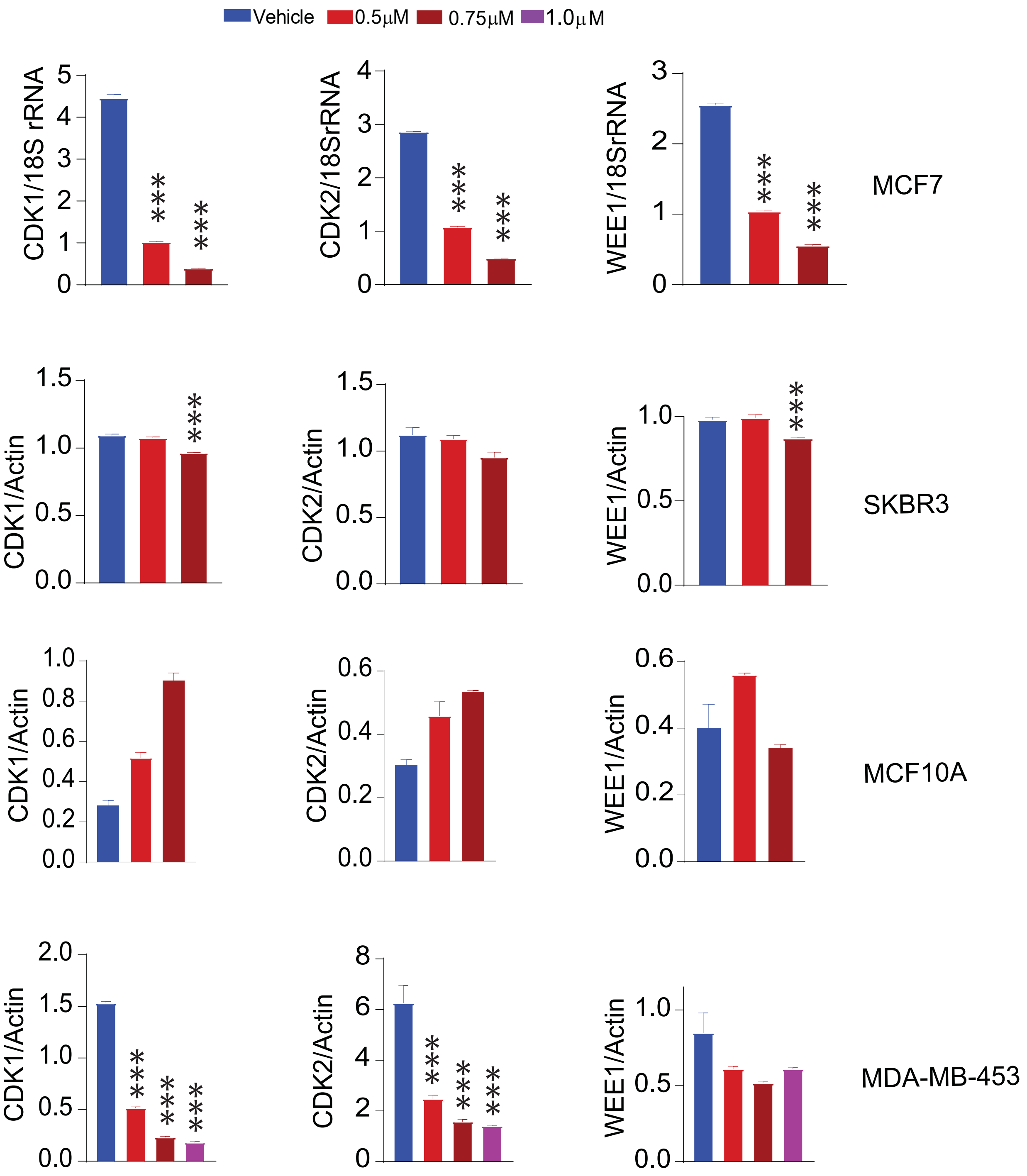

Supplementary Figure S9: Effect of ACK1 inhibition on CDKs and WEE1.

RNA from breast cancer cells treated with vehicle or varying concentrations of (*R*)-**9b** overnight were subjected to qRT-PCR using *CDK1*, *CDK2* and *WEE1* primers. *18S rRNA* was used as housekeeping control. Data are represented as the mean ± SEM from 2 biologically independent experiments. *$p<0.05$, **$p<0.01$, ***$p<0.001$, unpaired two-tailed Student's *t*-test.

Supplementary Table S1. Intensities of pY284-ACK1 and ACK1 in breast cancer TMA.

pY284-ACK1	ER+	PR+	ER+PR+	HER2+	TNBC
No Expression	15	1	19	8	18
Mild	24	2	61	14	32
Moderate	36	0	68	29	25
Strong	17	3	44	21	19
Total	92	6	192	72	94
ACK1	ER+	PR+	ER+PR+	HER2+	TNBC
No expression	55	5	143	48	51
Mild	18	0	32	9	21
Moderate	6	1	25	9	13
Strong	1	0	6	2	5
Total	80	6	206	68	90

Supplementary Table 2: Kinase Profiling Report for: Washington Univ. St. Louis

10 compounds tested against 1 kinase
Compounds were received as 10 mM stock.

Compounds were tested in 10-dose IC50 mode with a 3-fold serial dilution starting at 1 µM.
Control compound, staurosporine, was tested in 10-dose IC50 mode with 4-fold serial dilution starting at 20 µM.
Reactions were carried out at 1 µM ATP.

Data pages include raw data, % Enzyme activity (relative to DMSO controls) and curve fits.
***Curve fits were performed where the enzyme activities at the highest concentration of compounds were less than 65%.**

IC50 Summary:

	Compound IC_{50}* (M):	IC_{50} (nM)
Compound ID:	ACK1	ACK1
(R)-9b	1.25E-08	12.5 nM
1520	7.89E-08	78.9 nM
1503	1.92E-07	192.0 nM
1505	1.19E-07	119.0 nM
1506	7.67E-08	76.7 nM
1515	7.03E-08	70.3 nM
1517	9.87E-08	98.7 nM
STAUROSPORINE	5.60E-08	56.0 nM

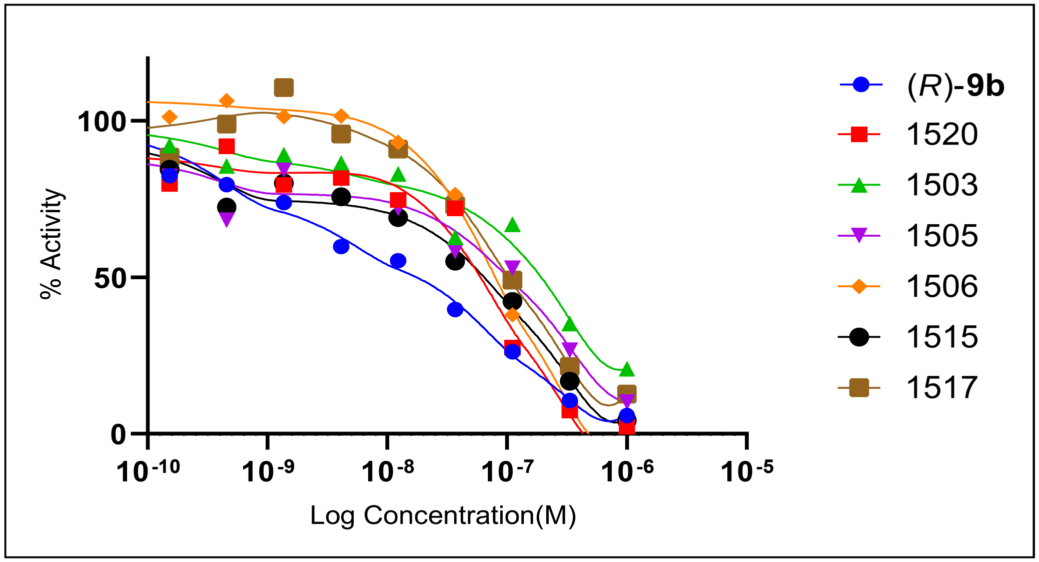

(R)-9b
1520
1503
1505
1506
1515
1517
% Activity
100
50
0
10^{-10} 10^{-9} 10^{-8} 10^{-7} 10^{-6} 10^{-5}
Log Concentration(M)

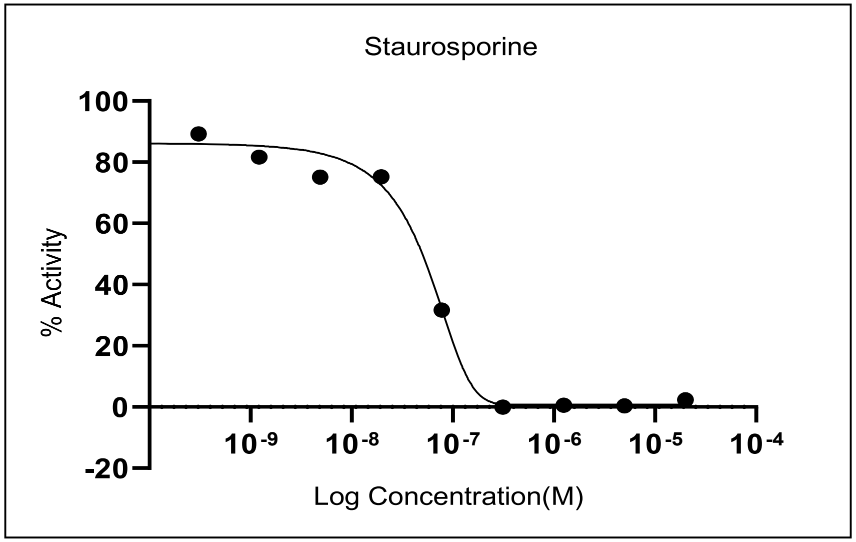

Staurosporine
% Activity
100
80
60
40
20
0
-20
10^{-9} 10^{-8} 10^{-7} 10^{-6} 10^{-5} 10^{-4}
Log Concentration(M)

Supplementary Table 3: Permeability of (*R*)-9bMS in Caco-2 Assay

Serial Number	Compound ID	Mean P_{app} (10^{-6} cm/s)		Efflux Ratio	Mean Recovery %		Rank		Note
		A to B	B to A		A to B	B to A	P_{app}	Efflux Transporter Substrate	
PC1	nadolol	0.10	ND	-	90.38	ND	Low	-	Low permeability marker
PC2	metoprolol	12.40	ND	-	92.27	ND	High	-	High permeability marker
PC3	Digoxin	0.02	9.60	435.85	94.27	96.22	Low	Likely	P-gp substrate
CPD1	**(R)-9bMS**	**22.59**	**19.18**	**0.85**	**73.62**	**107.51**	**High**	**Poor or non-**	**-**

ND: not determined

The permeation was assessed over a 120-minute incubation at 37 ± 1° C and 5% CO_2 with saturated humidity.

Binning Criteria*:

Low permeability: $P_{app} \leq 0.5$ (× 10^{-6} cm/s)

Moderate permeability:0.5 < P_{app} < 2.5 (× 10^{-6} cm/s)

High permeability: $P_{app} \geq 2.5$ (× 10^{-6} cm/s)

*The binning criteria of permeability are proposed based on WuXi routine Caco-2 permeability assay conditions (2 μM dosing concentration and 120 minutes incubation). The boundaries for low and high permeability binning are equivalent to 50% and 80% of the "calculated Fa" in human.

Supplementary Table S4: Cytochrome P450 (CYP) inhibition in human liver microsomes

Compound ID	IC_{50} (μM)				
	CYP1A2	CYP2C9	CYP2C19	CYP2D6	CYP3A4-M
(*R*)-9bMS	>50	>50	>50	>50	>50

Positive Controls

CYP Isozyme	Standard Inhibitor	*IC_{50} (μM)	IC_{50} Acceptance Range (μM)	Pass/No Pass
1A2	α-Naphthoflavone	0.218	0.125-0.448	Pass
2C9	Sulfaphenazole	0.593	0.333-0.750	Pass
2C19	(+)-N-3-benzylnirvanol	0.215	0.0928-0.333	Pass
2D6	Quinidine	0.140	0.0928-0.226	Pass
3A4	Ketoconazole	0.0446	0.0303-0.0928	Pass

Supplementary Table 5: (i) Protein binding results of (*R*)-9bMS and control compound in human plasma

Compound ID	Replicate	% Bound	% Unbound	LogK	% Recovery	% Remaining at 6 hr
warfarin	Replicate 1	98.53	1.47	1.83	92.14	96.06
	Replicate 2	98.53	1.47	1.83	92.53	97.85
	Mean	98.53	1.47	1.83	92.33	96.96
(*R*)-**9b**MS	Replicate 1	84.78	15.22	0.75	83.57	97.07
	Replicate 2	83.16	16.84	0.69	92.84	95.68
	Mean	83.97	16.03	0.72	88.21	96.37

(ii) Protein binding results of (*R*)-9bMS and control compound in rat plasma

Compound ID	Replicate	% Bound	% Unbound	LogK	% Recovery	% Remaining at 6 hr
warfarin	Replicate 1	99.31	0.69	2.16	86.73	88.24
	Replicate 2	99.29	0.71	2.15	87.38	90.55
	Mean	99.30	0.70	2.15	87.05	89.39
(*R*)-**9b**MS	Replicate 1	85.63	14.37	0.78	98.03	94.92
	Replicate 2	88.30	11.70	0.88	97.23	97.89
	Mean	86.97	13.03	0.83	97.63	96.41

Supplementary Table S6: (i) Stability of (*R*)-9b in Simulated Gastric Fluid (SGF)

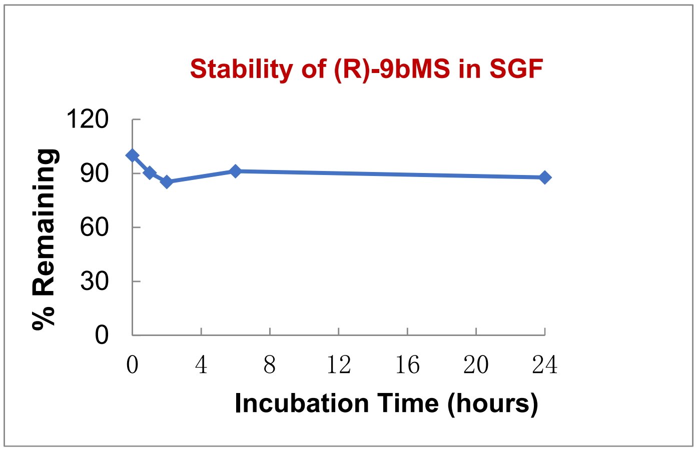

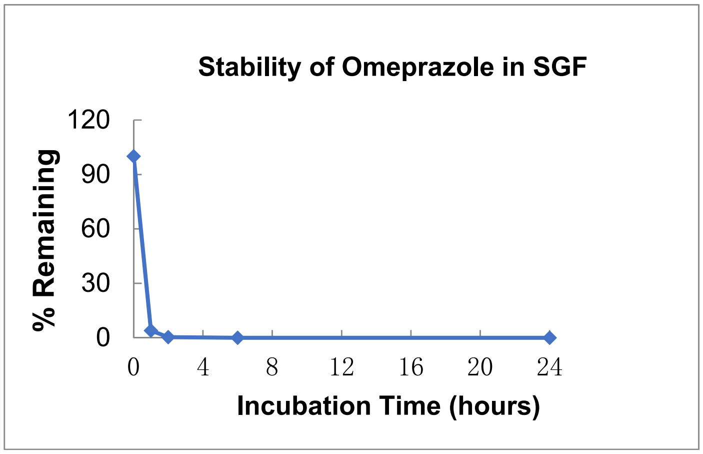

(ii) Stability of (R)-9b in Simulated Intestinal Fluid (SIF)

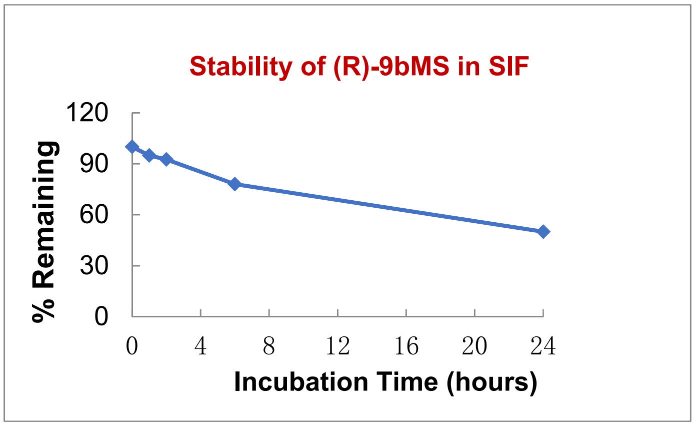

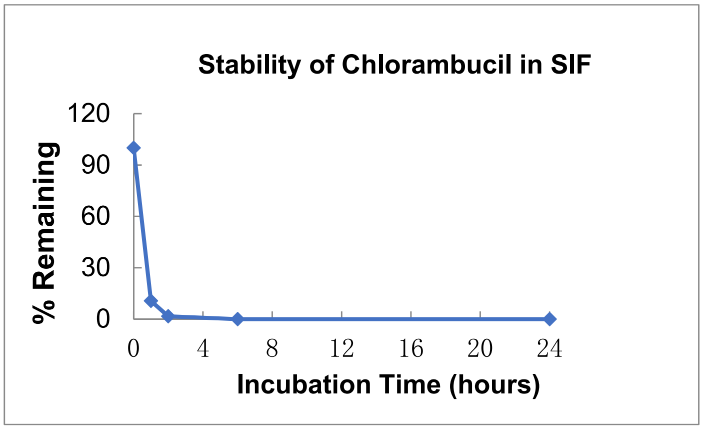

Supplementary Table 7

Compound Name	Target Class	Assay Name	Mode	Assay Target	ResultType	RC50(uM)	MaxResponse
(R)-9bMS	GPCR	Calcium Flux	Agonist	ADORA2A	EC50	10	0
(R)-9bMS	GPCR	Calcium Flux	Agonist	ADRA1A	EC50	10	2.3
(R)-9bMS	GPCR	Calcium Flux	Agonist	AVPR1A	EC50	10	10.08
(R)-9bMS	GPCR	Calcium Flux	Agonist	CCKAR	EC50	10	2.66
(R)-9bMS	GPCR	Calcium Flux	Agonist	CHRM1	EC50	10	0
(R)-9bMS	GPCR	Calcium Flux	Agonist	CHRM3	EC50	10	0
(R)-9bMS	GPCR	Calcium Flux	Agonist	EDNRA	EC50	10	2.42
(R)-9bMS	GPCR	Calcium Flux	Agonist	HRH1	EC50	10	1.97
(R)-9bMS	GPCR	Calcium Flux	Agonist	HTR2A	EC50	10	1.23
(R)-9bMS	GPCR	Calcium Flux	Agonist	HTR2B	EC50	0.08	66.18
(R)-9bMS	GPCR	Calcium Flux	Antagonist	ADORA2A	IC50	10	43.83
(R)-9bMS	GPCR	Calcium Flux	Antagonist	ADRA1A	IC50	10	13.13
(R)-9bMS	GPCR	Calcium Flux	Antagonist	AVPR1A	IC50	10	0
(R)-9bMS	GPCR	Calcium Flux	Antagonist	CCKAR	IC50	10	0
(R)-9bMS	GPCR	Calcium Flux	Antagonist	CHRM1	IC50	4.6	41.44
(R)-9bMS	GPCR	Calcium Flux	Antagonist	CHRM3	IC50	10	9.08
(R)-9bMS	GPCR	Calcium Flux	Antagonist	EDNRA	IC50	10	0
(R)-9bMS	GPCR	Calcium Flux	Antagonist	HRH1	IC50	10	0
(R)-9bMS	GPCR	Calcium Flux	Antagonist	HTR2A	IC50	10	2.95
(R)-9bMS	GPCR	Calcium Flux	Antagonist	HTR2B	IC50	0.4	95.05
(R)-9bMS	GPCR	cAMP	Agonist	ADRA2A	EC50	10	0.06
(R)-9bMS	GPCR	cAMP	Agonist	ADRB1	EC50	10	1.11
(R)-9bMS	GPCR	cAMP	Agonist	ADRB2	EC50	10	0.8
(R)-9bMS	GPCR	cAMP	Agonist	CHRM2	EC50	10	28.89
(R)-9bMS	GPCR	cAMP	Agonist	CNR1	EC50	10	0
(R)-9bMS	GPCR	cAMP	Agonist	CNR2	EC50	10	0
(R)-9bMS	GPCR	cAMP	Agonist	DRD1	EC50	10	0
(R)-9bMS	GPCR	cAMP	Agonist	DRD2S	EC50	10	5.4
(R)-9bMS	GPCR	cAMP	Agonist	HRH2	EC50	10	1.76
(R)-9bMS	GPCR	cAMP	Agonist	HTR1A	EC50	10	2.83
(R)-9bMS	GPCR	cAMP	Agonist	HTR1B	EC50	10	0
(R)-9bMS	GPCR	cAMP	Agonist	OPRD1	EC50	10	4.97
(R)-9bMS	GPCR	cAMP	Agonist	OPRK1	EC50	10	16.35
(R)-9bMS	GPCR	cAMP	Agonist	OPRM1	EC50	10	8.5
(R)-9bMS	GPCR	cAMP	Antagonist	ADRA2A	IC50	10	21.16
(R)-9bMS	GPCR	cAMP	Antagonist	ADRB1	IC50	10	9.71

**(R)-9bMS**	GPCR	cAMP	Antagonist	**ADRB2**	IC50	10	3.47
**(R)-9bMS**	GPCR	cAMP	Antagonist	**CHRM2**	IC50	10	9.27
**(R)-9bMS**	GPCR	cAMP	Antagonist	**CNR1**	IC50	10	3.21
**(R)-9bMS**	GPCR	cAMP	Antagonist	**CNR2**	IC50	10	30.79
**(R)-9bMS**	GPCR	cAMP	Antagonist	**DRD1**	IC50	10	11.31
**(R)-9bMS**	GPCR	cAMP	Antagonist	**DRD2S**	IC50	10	4.24
**(R)-9bMS**	GPCR	cAMP	Antagonist	**HRH2**	IC50	10	3.55
**(R)-9bMS**	GPCR	cAMP	Antagonist	**HTR1A**	IC50	10	5.39
**(R)-9bMS**	GPCR	cAMP	Antagonist	**HTR1B**	IC50	10	11.55
**(R)-9bMS**	GPCR	cAMP	Antagonist	**OPRD1**	IC50	10	0
**(R)-9bMS**	GPCR	cAMP	Antagonist	**OPRK1**	IC50	10	2.93
**(R)-9bMS**	GPCR	cAMP	Antagonist	**OPRM1**	IC50	10	14.33
**(R)-9bMS**	Ion Channel	Ion Channel	Blocker	**CAV1.2**	IC50	10	5.61
**(R)-9bMS**	Ion Channel	Ion Channel	Blocker	**GABAA**	IC50	10	17.26
**(R)-9bMS**	Ion Channel	Ion Channel	Blocker	**hERG**	IC50	10	28.29
**(R)-9bMS**	Ion Channel	Ion Channel	Blocker	**HTR3A**	IC50	9.6	50.28
**(R)-9bMS**	Ion Channel	Ion Channel	Blocker	**KvLQT1/minK**	IC50	10	17.72
**(R)-9bMS**	Ion Channel	Ion Channel	Blocker	**nAChR(a4/b2)**	IC50	3.3	44.02
**(R)-9bMS**	Ion Channel	Ion Channel	Blocker	**NAV1.5**	IC50	10	27.68
**(R)-9bMS**	Ion Channel	Ion Channel	Blocker	**NMDAR (1A/2B)**	IC50	10	5
**(R)-9bMS**	Ion Channel	Ion Channel	Opener	**GABAA**	EC50	10	0.79
**(R)-9bMS**	Ion Channel	Ion Channel	Opener	**HTR3A**	EC50	10	0.25
**(R)-9bMS**	Ion Channel	Ion Channel	Opener	**KvLQT1/minK**	EC50	10	1.08
**(R)-9bMS**	Ion Channel	Ion Channel	Opener	**nAChR(a4/b2)**	EC50	10	0
**(R)-9bMS**	Ion Channel	Ion Channel	Opener	**NMDAR (1A/2B)**	EC50	10	2.6
**(R)-9bMS**	Kinases	Binding	Inhibitor	**INSR**	IC50	0.18	99.32
**(R)-9bMS**	Kinases	Binding	Inhibitor	**LCK**	IC50	0.33	101.3
**(R)-9bMS**	Kinases	Binding	Inhibitor	**ROCK1**	IC50	0.16	100.08
**(R)-9bMS**	Kinases	Binding	Inhibitor	**VEGFR2**	IC50	0.22	99.23
**(R)-9bMS**	NHR	NHR Nuclear Translocation	Agonist	**AR**	EC50	10	0
**(R)-9bMS**	NHR	NHR Nuclear Translocation	Antagonist	**AR**	IC50	10	52.78
**(R)-9bMS**	NHR	NHR Protein Interaction	Agonist	**GR**	EC50	10	1.24
**(R)-9bMS**	NHR	NHR Protein Interaction	Antagonist	**GR**	IC50	10	20.2
**(R)-9bMS**	Non-Kinase Enzymes	Enzymatic	Inhibitor	**AChE**	IC50	10	30.95
**(R)-9bMS**	Non-Kinase Enzymes	Enzymatic	Inhibitor	**COX1**	IC50	1.2	105.71
**(R)-9bMS**	Non-Kinase Enzymes	Enzymatic	Inhibitor	**COX2**	IC50	1.5	106.79
**(R)-9bMS**	Non-Kinase Enzymes	Enzymatic	Inhibitor	**MAOA**	IC50	10	6.51
**(R)-9bMS**	Non-Kinase Enzymes	Enzymatic	Inhibitor	**PDE3A**	IC50	10	0

**(R)-9bMS**	Non-Kinase Enzymes	Enzymatic	Inhibitor	**PDE4D2**	IC50	10	11.73
**(R)-9bMS**	Transporter	Transporter	Blocker	**DAT**	IC50	10	24.62
**(R)-9bMS**	Transporter	Transporter	Blocker	**NET**	IC50	10	0
**(R)-9bMS**	Transporter	Transporter	Blocker	**SERT**	IC50	10	21.89

Control values

Compound Name	Target Class	Assay Name	Mode	Assay Target	ResultType	RC50(uM)
NECA	GPCR	Calcium Flux	Agonist	**ADORA2A**	EC50	0.01967
SCH 442416	GPCR	Calcium Flux	Antagonist	**ADORA2A**	IC50	0.04867
A-61603	GPCR	Calcium Flux	Agonist	**ADRA1A**	EC50	0.00005
Tamsulosin	GPCR	Calcium Flux	Antagonist	**ADRA1A**	IC50	0.0014
UK 14,304	GPCR	cAMP	Agonist	**ADRA2A**	EC50	0.00011
Yohimbine	GPCR	cAMP	Antagonist	**ADRA2A**	IC50	0.0085
Isoproterenol	GPCR	cAMP	Agonist	**ADRB1**	EC50	0.00133
Betaxolol	GPCR	cAMP	Antagonist	**ADRB1**	IC50	0.0046
Isoproterenol	GPCR	cAMP	Agonist	**ADRB2**	EC50	0.0011
ICI 118,551	GPCR	cAMP	Antagonist	**ADRB2**	IC50	0.00045
Vasopressin	GPCR	Calcium Flux	Agonist	**AVPR1A**	EC50	0.00034
SR 49059	GPCR	Calcium Flux	Antagonist	**AVPR1A**	IC50	0.00207
CCK-8	GPCR	Calcium Flux	Agonist	**CCKAR**	EC50	0.00065
SR 27897	GPCR	Calcium Flux	Antagonist	**CCKAR**	IC50	0.032
Acetylcholine	GPCR	Calcium Flux	Agonist	**CHRM1**	EC50	0.008
Atropine	GPCR	Calcium Flux	Antagonist	**CHRM1**	IC50	0.00387
Acetylcholine	GPCR	cAMP	Agonist	**CHRM2**	EC50	0.01133
Atropine	GPCR	cAMP	Antagonist	**CHRM2**	IC50	0.00647
Acetylcholine	GPCR	Calcium Flux	Agonist	**CHRM3**	EC50	0.018
Atropine	GPCR	Calcium Flux	Antagonist	**CHRM3**	IC50	0.00333
CP55940	GPCR	cAMP	Agonist	**CNR1**	EC50	0.00003
AM251	GPCR	cAMP	Antagonist	**CNR1**	IC50	0.0021
CP55940	GPCR	cAMP	Agonist	**CNR2**	EC50	0.00012
SR 144528	GPCR	cAMP	Antagonist	**CNR2**	IC50	0.02833
Dopamine	GPCR	cAMP	Agonist	**DRD1**	EC50	0.13333
SCH 39166	GPCR	cAMP	Antagonist	**DRD1**	IC50	0.00183
Dopamine	GPCR	cAMP	Agonist	**DRD2S**	EC50	0.00287
Risperidone	GPCR	cAMP	Antagonist	**DRD2S**	IC50	0.011
Endothelin 1	GPCR	Calcium Flux	Agonist	**EDNRA**	EC50	0.00072
BMS 182874	GPCR	Calcium Flux	Antagonist	**EDNRA**	IC50	0.73667
Histamine	GPCR	Calcium Flux	Agonist	**HRH1**	EC50	0.0037
Mepyramine	GPCR	Calcium Flux	Antagonist	**HRH1**	IC50	0.01173

Histamine	GPCR	cAMP	Agonist	**HRH2**	EC50	0.36
Tiotidine	GPCR	cAMP	Antagonist	**HRH2**	IC50	0.01633
Serotonin	GPCR	cAMP	Agonist	**HTR1A**	EC50	0.00413
Spiperone	GPCR	cAMP	Antagonist	**HTR1A**	IC50	0.028
Serotonin	GPCR	cAMP	Agonist	**HTR1B**	EC50	0.00015
SB 224289	GPCR	cAMP	Antagonist	**HTR1B**	IC50	0.012
Serotonin	GPCR	Calcium Flux	Agonist	**HTR2A**	EC50	0.0038
Altanserin	GPCR	Calcium Flux	Antagonist	**HTR2A**	IC50	0.01333
Serotonin	GPCR	Calcium Flux	Agonist	**HTR2B**	EC50	0.00243
LY 272015	GPCR	Calcium Flux	Antagonist	**HTR2B**	IC50	0.00091
DADLE	GPCR	cAMP	Agonist	**OPRD1**	EC50	0.00005
Naltriben	GPCR	cAMP	Antagonist	**OPRD1**	IC50	0.00035
Dynorphin A	GPCR	cAMP	Agonist	**OPRK1**	EC50	0.01833
nor-binaltorphimine	GPCR	cAMP	Antagonist	**OPRK1**	IC50	0.00557
DAMGO	GPCR	cAMP	Agonist	**OPRM1**	EC50	0.00102
Naloxone	GPCR	cAMP	Antagonist	**OPRM1**	IC50	0.00447
6a-Fluorotestosterone	NHR	NHR Nuclear Translocation	Agonist	**AR**	EC50	0.0018
Geldanamycin	NHR	NHR Nuclear Translocation	Antagonist	**AR**	IC50	0.03733
Dexamethasone	NHR	NHR Protein Interaction	Agonist	**GR**	EC50	0.04267
Mifepristone	NHR	NHR Protein Interaction	Antagonist	**GR**	IC50	0.04967
GBR 12909	Transporter	Transporter	Blocker	**DAT**	IC50	0.00607
Desipramine	Transporter	Transporter	Blocker	**NET**	IC50	0.00937
Clomipramine	Transporter	Transporter	Blocker	**SERT**	IC50	0.002
Isradipine	Ion Channel	Ion Channel	Blocker	**CAV1.2**	IC50	0.0088
GABA	Ion Channel	Ion Channel	Opener	**GABAA**	EC50	5.76667
Picrotoxin	Ion Channel	Ion Channel	Blocker	**GABAA**	IC50	2.3
astemizole	Ion Channel	Ion Channel	Blocker	**hERG**	IC50	0.096
Serotonin	Ion Channel	Ion Channel	Opener	**HTR3A**	EC50	0.21667
Bemesetron	Ion Channel	Ion Channel	Blocker	**HTR3A**	IC50	0.00177
ML-277	Ion Channel	Ion Channel	Opener	**KvLQT1/minK**	EC50	2.9
XE 991	Ion Channel	Ion Channel	Blocker	**KvLQT1/minK**	IC50	0.94667
(-)-Nicotine	Ion Channel	Ion Channel	Opener	**nAChR(a4/b2)**	EC50	0.59
Dihydro-ß-erythroidine	Ion Channel	Ion Channel	Blocker	**nAChR(a4/b2)**	IC50	0.73
Lidocaine	Ion Channel	Ion Channel	Blocker	**NAV1.5**	IC50	32.66667

L-Glutamic Acid	Ion Channel	Ion Channel	Opener	**NMDAR (1A/2B)**	EC50	0.41333
(+)-MK 801	Ion Channel	Ion Channel	Blocker	**NMDAR (1A/2B)**	IC50	0.01027
Physostigmine	Non-Kinase Enzymes	Enzymatic	Inhibitor	**AChE**	IC50	0.15
Indomethacin	Non-Kinase Enzymes	Enzymatic	Inhibitor	**COX1**	IC50	0.05067
NS-398	Non-Kinase Enzymes	Enzymatic	Inhibitor	**COX2**	IC50	0.08267
Clorgyline	Non-Kinase Enzymes	Enzymatic	Inhibitor	**MAOA**	IC50	0.0022
Cilostamide	Non-Kinase Enzymes	Enzymatic	Inhibitor	**PDE3A**	IC50	0.033
Cilomilast	Non-Kinase Enzymes	Enzymatic	Inhibitor	**PDE4D2**	IC50	0.01467
BMS-754807	Kinases	Binding	Inhibitor	**INSR**	IC50	0.00066
Gleevec	Kinases	Binding	Inhibitor	**LCK**	IC50	0.09033
Staurosporine	Kinases	Binding	Inhibitor	**ROCK1**	IC50	0.00023
Sunitinib	Kinases	Binding	Inhibitor	**VEGFR2**	IC50	0.0004

Supplementary Table S8. Cell proliferation regression analysis of breast cell lines

[Inhibitor] vs. response -- Variable slope (four parameters)									
	HCC-1395	MDA-MB-231	T47D	SKBR3	Cal-148	MDA-MB-453	MCF7	MCF10a	4T1
Best-fit values									
Bottom	-5.70	0.99	0.93	1.40	-7.84	-138.20	18.10	18.37	-0.01
Top	91.64	91.17	90.22	77.74	91.74	87.35	115.8	103.7	94.25
IC_{50}	1.22	0.41	0.3	0.66	0.63	0.84	0.45	1.49	0.25
Goodness of Fit									
R squared	0.895	0.937	0.930	0.899	0.897	0.894	0.856	0.764	0.976

Supplementary Table S9. Genes downregulated upon ACK1 inhibition.

Gene name	Fold Decrease
CDC20	170.48
CDCA8	28.282
CCNG1	22.864
CCNA2	20.248
CKS2	17.599
CXCR4	12.232
CDK13	10.935
CLK4	10.866
CLK3	10.696
CDK11B	10.683
CDCA4	10.04
CCNB2	9.8742
CCNT1	8.8911
CCNF	8.5853
CDC34	8.4848
CLK1	7.4731
CCNB1	7.3608
CDC6	7.0042
CDC27	5.6831

Supplementary Table 10

Supplementary Table 10 uploaded separately

Supplementary Table S11:

A. Gene Annotated Peaks in Vehicle and (*R*)-9b treated sample

	Vehicle	(R)-9b
**3'UTR**	11	
**5'UTR**	1	
**exon**	7	3
**Intergenic**	460	41
**intron**	428	18
**non-coding**	6	
**promoter-TSS**	11	5
**TTS**	11	1

B. Differentially modulated gene types in Vehicle and (*R*)-9b treated samples

	Vehicle	(R)-9b
**ncRNA**	262	18
**Protein-coding**	612	32
**Pseudo**	45	15
**rRNA**	3	2
**snoRNA**	7	
**snRNA**	1	1

Supplementary Table S12: List of primers

**ChIP Primers**	
CCNB1 Forward Primer	TTTAACCCAGGAGCAGGCAG
CCNB1 Reverse Primer	CTGGTCCACTCCCTTCCAAA
CCNB2 Forward Primer	TCTCTCTGCCTGCCTGTCTC
CCNB2 Reverse Primer	CTGTGCACTCAAGTGGCATT
CDC20 Forward Primer	CCTTGGCTATATGTCATGCCCACA
CDC20 Reverse Primer	AAGCCATGGCCTGAGATGAGC
CXCR4 Forward Primer	TTT GTT GGC TGC GGC AGC AGG
CXCR4 Reverse Primer	TTT TGG AGT ACG GGT ACC TCC
**qRT-PCR Primers**	
CCNB1 Forward Primer	GACCTGTGTCAGGCTTTCTCTG
CCNB1 Reverse Primer	GGTATTTTGGTCTGACTGCTTGC
CCNB2 Forward Primer	CAACCAGAGCAGCACAAGTAGC
CCNB2 Reverse Primer	GGAGCCAACTTTTCCATCTGTAC
CDC20 Forward Primer	CGGAAGACCTGCCGTTACATTC
CDC20 Reverse Primer	CAGAGCTTGCACTCCACAGGTA
ACK1 Forward Primer	ACTTTGGGCTGATGCGAGCACT
ACK1 Reverse Primer	AAGGTGCGTGTCTTCAGGCTCT
CXCR4 Forward Primer	CTCCTCTTTGTCATCACGCTTCC
CXCR4 Reverse Primer	GGATGAGGACACTGCTGTAGAG
18S rRNA Forward Primer	GGCCCTGTAATTGGAATGAGTC
18S rRNA Reverse Primer	CCAAGATCCAACTACGAGCTT
